# HIV incidence after pre-exposure prophylaxis initiation among women and men at elevated HIV risk: A population-based study in rural Kenya and Uganda

**DOI:** 10.1371/journal.pmed.1003492

**Published:** 2021-02-09

**Authors:** Catherine A. Koss, Diane V. Havlir, James Ayieko, Dalsone Kwarisiima, Jane Kabami, Gabriel Chamie, Mucunguzi Atukunda, Yusuf Mwinike, Florence Mwangwa, Asiphas Owaraganise, James Peng, Winter Olilo, Katherine Snyman, Benard Awuonda, Tamara D. Clark, Douglas Black, Joshua Nugent, Lillian B. Brown, Carina Marquez, Hideaki Okochi, Kevin Zhang, Carol S. Camlin, Vivek Jain, Monica Gandhi, Craig R. Cohen, Elizabeth A. Bukusi, Edwin D. Charlebois, Maya L. Petersen, Moses R. Kamya, Laura B. Balzer

**Affiliations:** 1 Division of HIV, Infectious Diseases, and Global Medicine, University of California, San Francisco, San Francisco, California, United States of America; 2 Centre for Microbiology Research, Kenya Medical Research Institute, Nairobi, Kenya; 3 Infectious Diseases Research Collaboration, Kampala, Uganda; 4 Department of Biostatistics and Epidemiology, University of Massachusetts, Amherst, Amherst, Massachusetts, United States of America; 5 Department of Obstetrics, Gynecology and Reproductive Sciences, University of California, San Francisco, San Francisco, California, United States of America; 6 Division of Prevention Science, Department of Medicine, University of California, San Francisco, San Francisco, California, United States of America; 7 Graduate Group in Biostatistics, School of Public Health, University of California, Berkeley, Berkeley, California, United States of America; 8 School of Medicine, Makerere University College of Health Sciences, Kampala, Uganda; University of Southampton, UNITED KINGDOM

## Abstract

**Background:**

Oral pre-exposure prophylaxis (PrEP) is highly effective for HIV prevention, but data are limited on HIV incidence among PrEP users in generalized epidemic settings, particularly outside of selected risk groups. We performed a population-based PrEP study in rural Kenya and Uganda and sought to evaluate both changes in HIV incidence and clinical and virologic outcomes following seroconversion on PrEP.

**Methods and findings:**

During population-level HIV testing of individuals ≥15 years in 16 communities in the Sustainable East Africa Research in Community Health (SEARCH) study (NCT01864603), we offered universal access to PrEP with enhanced counseling for persons at elevated HIV risk (based on serodifferent partnership, machine learning–based risk score, or self-identified HIV risk). We offered rapid or same-day PrEP initiation and flexible service delivery with follow-up visits at facilities or community-based sites at 4, 12, and every 12 weeks up to week 144. Among participants with incident HIV infection after PrEP initiation, we offered same-day antiretroviral therapy (ART) initiation and analyzed HIV RNA, tenofovir hair concentrations, drug resistance, and viral suppression (<1,000 c/ml based on available assays) after ART start. Using Poisson regression with cluster-robust standard errors, we compared HIV incidence among PrEP initiators to incidence among propensity score–matched recent historical controls (from the year before PrEP availability) in 8 of the 16 communities, adjusted for risk group. Among 74,541 individuals who tested negative for HIV, 15,632/74,541 (21%) were assessed to be at elevated HIV risk; 5,447/15,632 (35%) initiated PrEP (49% female; 29% 15–24 years; 19% in serodifferent partnerships), of whom 79% engaged in ≥1 follow-up visit and 61% self-reported PrEP adherence at ≥1 visit. Over 7,150 person-years of follow-up, HIV incidence was 0.35 per 100 person-years (95% confidence interval [CI] 0.22–0.49) among PrEP initiators. Among matched controls, HIV incidence was 0.92 per 100 person-years (95% CI 0.49–1.41), corresponding to 74% lower incidence among PrEP initiators compared to matched controls (adjusted incidence rate ratio [aIRR] 0.26, 95% CI 0.09–0.75; *p* = 0.013). Among women, HIV incidence was 76% lower among PrEP initiators versus matched controls (aIRR 0.24, 95% CI 0.07–0.79; *p* = 0.019); among men, HIV incidence was 40% lower, but not significantly so (aIRR 0.60, 95% CI 0.12–3.05; *p* = 0.54). Of 25 participants with incident HIV infection (68% women), 7/25 (28%) reported taking PrEP ≤30 days before HIV diagnosis, and 24/25 (96%) started ART. Of those with repeat HIV RNA after ART start, 18/19 (95%) had <1,000 c/ml. One participant with viral non-suppression was found to have transmitted viral resistance, as well as emtricitabine resistance possibly related to PrEP use. Limitations include the lack of contemporaneous controls to assess HIV incidence without PrEP and that plasma samples were not archived to assess for baseline acute infection.

**Conclusions:**

Population-level offer of PrEP with rapid start and flexible service delivery was associated with 74% lower HIV incidence among PrEP initiators compared to matched recent controls prior to PrEP availability. HIV infections were significantly lower among women who started PrEP. Universal HIV testing with linkage to treatment and prevention, including PrEP, is a promising approach to accelerate reductions in new infections in generalized epidemic settings.

**Trial registration:**

ClinicalTrials.gov NCT01864603.

## Abstract 2

Catherine Koss and co-workers report on HIV incidence in African settings where antiretroviral pre-exposure prophylaxis has been implemented.

## Author summary

**Why was this study done?:**

- Despite major gains in HIV testing and treatment, there were 1.7 million new HIV infections worldwide in 2019, of which nearly 60% occurred in sub-Saharan Africa.
- Daily oral pre-exposure prophylaxis (PrEP) with tenofovir disoproxil fumarate/emtricitabine (TDF/FTC) is highly effective for HIV prevention and could substantially reduce new HIV infections if offered alongside access to HIV testing and treatment.
- Little is known about the incidence of new HIV infections among PrEP users in settings with generalized HIV epidemics, particularly when offered broadly across communities where access to HIV testing and treatment have already been scaled up.

**What did the researchers do and find?:**

- In 16 communities in rural Kenya and Uganda, we conducted community-wide HIV testing and offered universal access to PrEP with an inclusive approach to eligibility (for persons in serodifferent partnerships, those identified by an HIV risk prediction tool, or those who self-identified as being at risk of HIV). We offered rapid PrEP start and a flexible care delivery model with follow-up visits at health facilities or community-based sites for HIV testing and PrEP refills.
- Of 74,541 persons who tested negative for HIV, 15,632 (21%) were assessed to be at elevated HIV risk, of whom 5,447 (35%) started PrEP. Overall, 79% of persons who initiated PrEP engaged in the program for follow-up visits.
- Among PrEP initiators in the 16 study communities, there were 25 seroconversions over 7,150 person-years of follow-up. HIV incidence was 0.35 per 100 person-years (95% confidence interval [CI] 0.22–0.49).
- In 8 communities, we compared HIV incidence among PrEP initiators to persons with similar characteristics (matched controls) from the year before PrEP was available. Compared to matched controls, HIV incidence was 74% lower among PrEP initiators overall; 76% lower among women who initiated PrEP; and 40% lower among men who initiated PrEP, although this result among men did not reach statistical significance.

**What do these findings mean?:**

- Providing universal access to PrEP in the context of community-wide HIV testing in rural Kenya and Uganda was associated with lower HIV incidence among persons who initiated PrEP compared to matched recent controls.
- We found lower HIV incidence after PrEP initiation among women, for whom rates of new HIV infections are higher than in men, including in recent prevention studies without PrEP. These results suggest that PrEP may help to close the gap in new infections between men and women.
- Universal access to HIV testing, treatment, and prevention, including rapid provision of PrEP with flexible service delivery, could reduce HIV incidence in generalized epidemic settings.

## Introduction

In 2019, there were 1.7 million new HIV infections globally, far exceeding the UNAIDS 2020 target of 500,000 new infections annually [1]. Oral pre-exposure prophylaxis (PrEP) with tenofovir disoproxil fumarate/emtricitabine (TDF/FTC) is highly effective for HIV prevention [2,3] and could accelerate reductions in HIV incidence in combination with other approaches such as treatment as prevention [4]. In high-income settings where both antiretroviral therapy (ART) and PrEP have been scaled up, declines in incidence have been observed among men who have sex with men (MSM) [5,6]. In sub-Saharan Africa, which accounted for 59% of new infections globally in 2019 [1], there have been remarkable gains in ART coverage, but PrEP rollout is just beginning to expand in many settings. As a result, data on HIV incidence among PrEP users in Africa remain limited outside of placebo-controlled trials and open-label studies of specific risk groups, such as serodifferent couples [7], young women [8], MSM [9], and female sex workers [10].

As PrEP is scaled up in generalized epidemic settings, optimal strategies for delivery (including care provision, refills, and laboratory monitoring) are needed to realize its potential to reduce new HIV infections. Community-based HIV testing with supported linkage to treatment or prevention, including PrEP, provides an opportunity to engage individuals (such as adolescent girls, young adults, and men) who may not otherwise access health services. Moreover, lower-barrier models for PrEP service delivery, including options for out-of-facility community-based visits, may further help to enhance retention of individuals in HIV prevention services.

We offered universal access to PrEP with an inclusive approach to eligibility during population-level HIV testing in 16 communities in rural Kenya and Uganda. We provided rapid PrEP start on-site at health fairs and at clinics and a flexible delivery model with follow-up visits at either clinics or community-based sites for both HIV testing and PrEP refills. We sought to evaluate HIV incidence among PrEP initiators and characterize incident HIV infections on PrEP, including clinical and virologic outcomes.

## Methods

### Ethics statement

This study was approved by the institutional review boards of Makerere University (Kampala, Uganda), Kenya Medical Research Institute (Nairobi, Kenya), and University of California, San Francisco (UCSF; San Francisco, California, United States of America). All participants provided verbal consent; PrEP participants provided written informed consent in their preferred language. This study is reported as per the STROBE Statement (S1 STROBE Checklist).

### Study design and procedures

The Sustainable East Africa Research in Community Health (SEARCH) study (NCT01864603) is a cluster-randomized controlled trial in 32 communities in rural Kenya and Uganda that began in 2013 to test the hypothesis that HIV “test and treat” with universal ART using a multi-disease, patient-centered care model would reduce new HIV infections and improve community health compared to a country guideline approach [11]. In 2016 to 2017, the study implemented a population-level PrEP intervention in 16 communities before national PrEP rollout in Kenya and Uganda. As previously described [12,13], from 2016 to 2017, we conducted community sensitization and education on PrEP and offered universal access to PrEP during population-level HIV and multi-disease testing, using a hybrid mobile testing approach [14]. Our approach involved holding health fairs at multiple locations across each community over 2 weeks, followed by home-based testing for non-attendees. We offered enhanced individual counseling on PrEP to persons with elevated risk of HIV acquisition based on at least one of the following categories: persons in serodifferent partnerships; those classified as being at risk based on an empirical HIV risk prediction algorithm developed using machine learning [15]; and individuals who self-identified as being at risk [12]. We offered rapid or same-day PrEP initiation (with medication provided by the study) at local government clinics (with one-time, study-provided transport). In 14 of 16 communities, on-site PrEP start was also offered at SEARCH community-wide health fairs. From 2017 to 2018, we also offered on-site PrEP initiation during HIV testing events for key populations, tailored to the epidemiology of each community and including groups such as serodifferent partners, young women, and persons working in the fishing or transportation industries, or in bars [16]. In addition, PrEP initiation was offered through the SEARCH study on an ongoing basis at clinics in study communities.

PrEP eligibility criteria included negative HIV testing within the preceding 4 weeks, no known hepatitis B infection, and no acute HIV symptoms. Baseline creatinine testing was performed, but PrEP was provided at enrollment before the receipt of creatinine results. After providing written informed consent, participants were given TDF (300 mg) co-formulated with FTC (200 mg). In a small number of cases, TDF co-formulated with lamivudine (3TC; 150 mg) was provided as an alternative to TDF/FTC due to limitations in drug supply, in accordance with guidelines [17,18].

Follow-up visits were scheduled at week 4, week 12, and every 12 weeks thereafter for up to 144 weeks prior to referral to local clinics for ongoing care. We provided a flexible delivery system with options for follow-up visits at clinics or community locations of the participants’ choice (e.g., homes, near schools, trading centers, or beaches). Follow-up visit procedures included evaluation of self-assessed HIV risk, self-reported PrEP adherence using 3-day recall [19] (a feasible-to-collect measure), rapid HIV antibody testing using country-standard serial testing algorithms, and PrEP refills. Participants who stopped PrEP were offered HIV testing and the opportunity to restart PrEP at each visit.

### Procedures for participants with incident HIV infection after PrEP initiation

Same-day ART start with standard regimens (TDF and 3TC with efavirenz [EFV] or, later, dolutegravir) recommended in Ugandan [20] and Kenyan [17,21] treatment guidelines was offered at the seroconversion visit (i.e., on the day of positive testing with 2 rapid antibody tests). Confirmatory testing with HIV RNA was performed, followed by testing with Geenius HIV 1/2 Supplemental Assay (Bio-Rad, Hercules, California, USA) or western blot if HIV RNA not detected. Resistance to antiretroviral drugs was assessed by standard consensus sequencing from a stored plasma sample collected at the seroconversion visit or the visit closest to the seroconversion visit. Among participants who self-reported taking PrEP ≤30 days prior to seroconversion, we analyzed tenofovir concentrations in small hair samples (50 to 100 strands) to estimate the number of PrEP doses taken per week [22,23]. One centimeter of hair closest to the scalp (reflecting the most recent 4 weeks of drug exposure) was analyzed via liquid chromatography-tandem mass spectrometry (LC-MS/MS) using validated methods in the UCSF Hair Analytical Laboratory [22]. Among participants with incident HIV infection, we assessed viral suppression rates ≤12 months after ART start. A threshold for viral suppression of HIV RNA <1,000 copies/ml was selected based on the highest limit of detection of assays used during routine follow-up (ranging from <20 to <1,000 copies/ml).

### Statistical analysis

We analyzed the following steps of the PrEP cascade, based on methods previously described [12], with data updated in this analysis through database closure (June 17, 2020). Among individuals assessed to be at elevated HIV risk, we calculated the proportion with PrEP uptake, defined as initiation based on receipt of pills. Among individuals who initiated PrEP, we measured program engagement, defined as attendance at follow-up visits. At each follow-up visit, we assessed the proportion of individuals who (1) received PrEP medication refills; and (2) self-reported adherence to PrEP (at least 1 dose of the past 3 [an indication of any recent PrEP use], making the assumption that the participants not seen were nonadherent), both among all individuals who initiated PrEP and among individuals who reported current HIV risk.

We analyzed the HIV incidence rate among PrEP initiators who had repeat HIV testing after PrEP initiation. Incident HIV infections were those confirmed by HIV RNA, western blot, or Geenius testing. The date of HIV infection was imputed as the midpoint between last negative and first positive test. Follow-up time was censored at the date of death, last visit before database closure, or the imputed infection date. Incidence rates were calculated overall and by sex with 95% confidence intervals (CIs) defined by 2.5th and 97.5th quantiles over 5,000 bootstrap samples.

We conducted an analysis to compare observed HIV incidence among PrEP initiators to expected HIV incidence without PrEP. Because a contemporaneous control group without access to PrEP and with repeat HIV testing to detect seroconversions was not available, we compared HIV incidence among PrEP initiators to HIV incidence among propensity score–matched recent controls over the year before PrEP was available through this study. This analysis was thus restricted to the 8 study communities in which population-level HIV testing was performed (as described above [14]) 1 year before PrEP was available and was repeated 1 year later at the start of the PrEP intervention. Individuals in these communities who had a negative HIV test in 2015 to 2016 and had a repeat HIV test 1 year later (2016 to 2017) were eligible to contribute to the analysis of matched controls. The dates of population-level HIV testing in the 8 communities are provided in S1 Table. (In the 8 communities not included in this analysis, population-level HIV testing was not performed the year prior to PrEP availability and, thus, HIV incidence data were not available). Because PrEP is intended for use among persons at elevated HIV risk, we selected recent historical controls based on 1-to-1 matching on an estimated propensity score, with matching performed within each community. Specifically, the propensity score was defined as the conditional probability of PrEP initiation given the following HIV risk predictors [24]: demographic factors, including age, sex, occupation, education, mobility, alcohol use, and serodifferent partnership (full list in S1 Statistical Analysis Plan). We estimated the propensity score with the machine learning algorithm Super Learner [25], using 5-fold interval cross validation. We then selected among the recent controls based on matching (via the *Matching* package [24] in R) on the estimated propensity score. Finally, we calculated incidence rate ratios using Poisson regression with robust standard errors, accounting for clustering by community. Regression models adjusted for risk group (serodifferent partners, women 15 to 24 years, widow[er]s, fishing/bar/transport workers, and alcohol users) to account for any residual differences between the risk profiles of PrEP initiators and the matched controls. To examine differences by sex, these analyses were repeated stratifying on sex. Additional details are available in S1 Statistical Analysis Plan. Analyses were conducted using R version 3.6.1.

## Results

### Study participants and PrEP uptake

**Fig 1:**
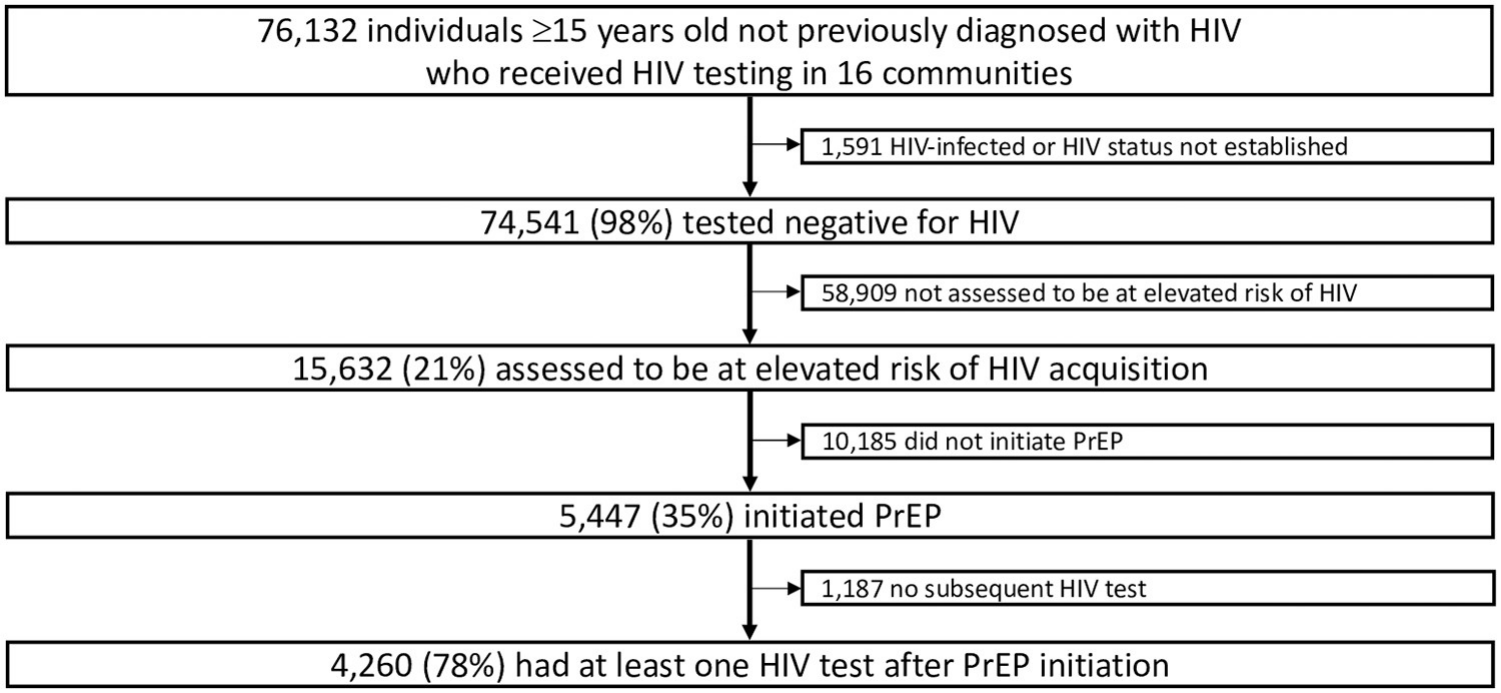
PrEP uptake following population-level HIV testing in 16 communities in rural Kenya and Uganda. Individuals assessed to be at elevated risk of HIV acquisition (based on serodifferent partnership, HIV risk score, or otherwise self-identified HIV risk) were offered enhanced individual counseling on PrEP. Individuals neither in serodifferent partnerships nor identified by the risk score could self-identify as at risk of HIV acquisition. PrEP, pre-exposure prophylaxis.

A total of 76,132 individuals ≥15 years old not previously diagnosed with HIV received HIV testing in the 16 study communities (Fig 1) from June 2016 to April 2019. Overall, 74,541 tested negative for HIV, of whom 15,632 (21%) were assessed to be at elevated HIV risk, and 5,447 (35%) initiated PrEP (S2 Table). Among the 5,447 PrEP initiators, 49% were women, 29% were age 15 to 24 years, 16% were age ≥45 years, and 19% were in serodifferent partnerships (Table 1; S3 Table).

**Table 1:** Baseline characteristics of individuals who initiated PrEP in 16 communities in rural Kenya and Uganda.

		PrEP initiators (*N* = 5,447)
Sex	Female	2,674 (49.1%)
	Male	2,773 (50.9%)
Age, years	15–24	1,582 (29.0%)
	25–34	1,879 (34.5%)
	35–44	1,125 (20.6%)
	45–54	600 (11.0%)
	≥55	261 (4.8%)
Educational attainment^a^	Less than primary level	292 (5.4%)
	Primary school level	3,279 (60.2%)
	Any secondary school level or higher	1,213 (22.3%)
Occupation^b^	Farmer	2,330 (42.8%)
	Student	247 (4.5%)
	Fishing, bar, or transportation	1,102 (20.2%)
	Other informal sector	981 (18.0%)
	Other formal sector	203 (3.7%)
	Unemployed or disabled	218 (4.0%)
	Other or unknown	19 (0.3%)
Marital status^c^	Not married	1,053 (19.3%)
	Married (monogamous)	2,618 (48.1%)
	Married (polygamous)	960 (17.6%)
	Divorced, separated, or widowed	469 (8.6%)
Serodifferent partner	Yes	1,026 (18.8%)
	No or unknown	4,421 (81.2%)
Circumcision^d^	Medical	742 (26.8%)
	Traditional	452 (16.3%)
	Uncircumcised	1,241 (44.8%)
Alcohol use^e^	None	3,896 (71.5%)
	1–7 days per month	357 (6.6%)
	>7 days per month	536 (9.8%)
Mobility^f^	Yes	315 (5.8%)
	No	4,751 (87.2%)
Region	Western Kenya	2,413 (44.3%)
	Eastern Uganda	1,471 (27.0%)
	Western Uganda	1,563 (28.7%)

^a^Missing data for 663 (12.2%) individuals.

^b^Other formal sector occupations: teaching, government, military, healthcare, and factory work. Other informal sector occupations: shopkeeper, market vendor, hotel worker, homemaker, household worker, miner, and construction. Missing data for 347 (6.4%) individuals.

^c^Missing data for 347 (6.3%) individuals.

^d^Assessed among 2,773 men. Missing data for 338 (12.2%) individuals.

^e^Missing data for 658 (12.1%) individuals.

^f^Mobility defined as migration out of the community for at least 1 month or moved residence within the past 12 months. Missing data for 381 (7.0%) individuals.

PrEP, pre-exposure prophylaxis.

### PrEP program engagement, refills, and self-reported adherence

Among 5,398 PrEP initiators eligible for a follow-up visit, 4,271 (79%) engaged in the PrEP program and attended ≥1 follow-up visit, 3,578 (66%) received ≥1 refill, and 3,282 (61%) self-reported adherence to PrEP at ≥1 visit. At week 4, 3,512/5,398 (65%) individuals who initiated PrEP were engaged in the PrEP program, 2,805 (52%) received PrEP refills, and 2,271 (42%) self-reported adherence to PrEP (at least 1 dose of the last 3) (S1 Fig). At week 60, 2,758 (54%) of 5,094 eligible participants were engaged in the program, 1,677 (33%) received a refill, and 1,367 (27%) self-reported adherence. Among participants who reported current HIV risk at follow-up visits, refills and self-reported adherence were higher. At week 60, of the 1,711 participants engaged and reporting current HIV risk, 1,601 (94%) received refills and 1,277 (75%) self-reported adherence. Overall, 83% of PrEP initiators stopped PrEP at least once and 45% of those later restarted PrEP. Among the 5,447 participants who started PrEP, 145 (2.7%) received at least 1 fill of TDF/3TC (rather than TDF/FTC).

In analyses stratified by sex, women were more likely than men to engage in PrEP visits, receive refills, and report adherence at each study visit through week 60 (S2 Fig). Moreover, throughout the study, women were more likely than men to ever engage in visits, receive refills, or report adherence (82%, 69%, and 65%, respectively, among women compared to 75%, 62%, and 56% among men). In analyses by age–sex strata, at week 24, youth ages 15 to 24 years were less likely to engage in all steps of the PrEP cascade and to report current HIV risk compared to older groups (S3 Fig).

### HIV incidence among PrEP initiators

**Fig 2:**
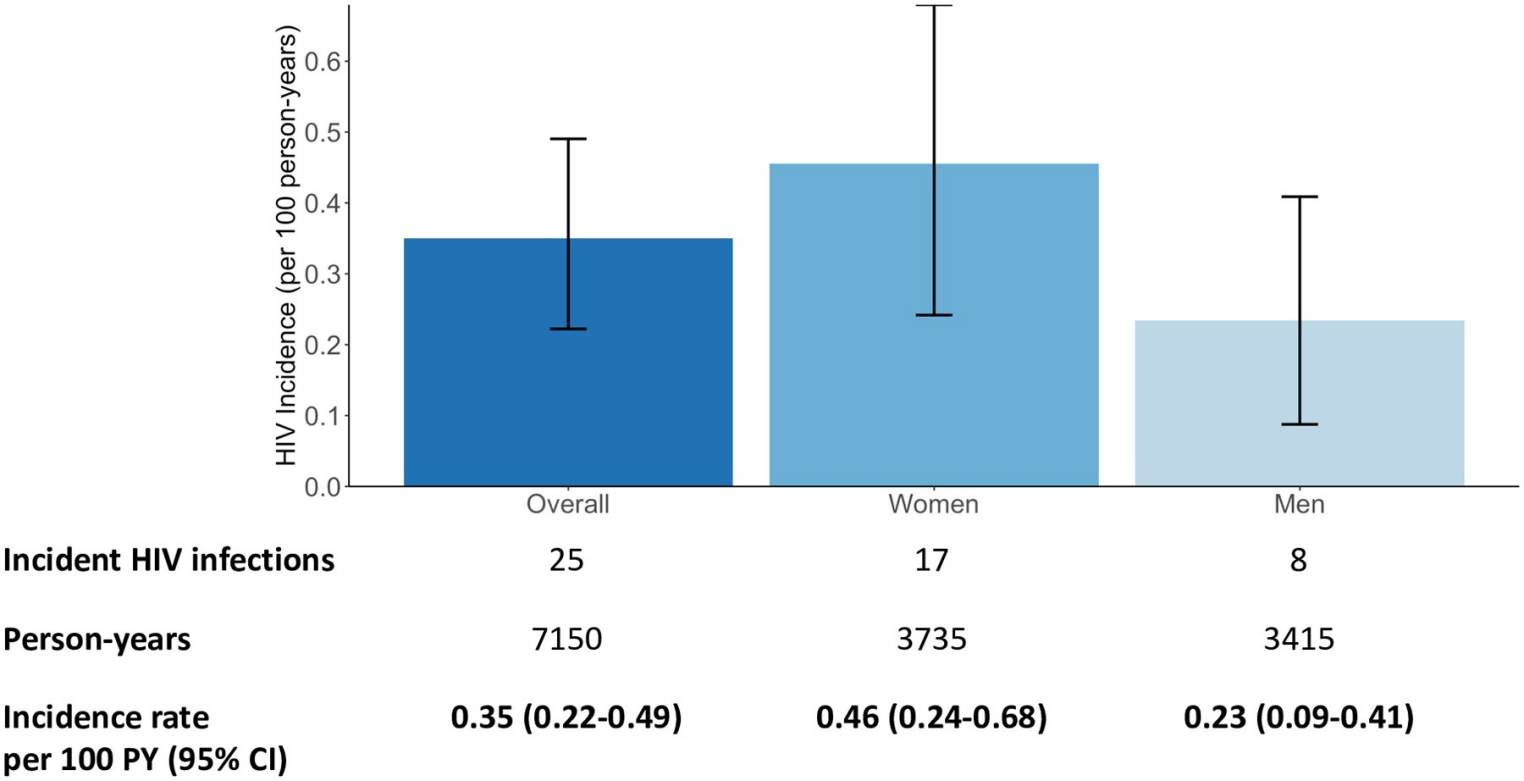
Observed HIV incidence rate among PrEP initiators, overall and stratified by sex. Observed HIV incidence rate per 100 person-years among PrEP initiators in all 16 study communities. CI, confidence interval; PrEP, pre-exposure prophylaxis; PY, person-years.

Among 5,447 PrEP initiators, 4,260 (78%) had at least 1 subsequent HIV test after PrEP initiation (S4 Table). There were 25 incident HIV infections over 7,150 person-years of follow-up. The HIV incidence rate was 0.35 per 100 person-years (95% CI 0.22 to 0.49) overall, 0.46 per 100 person-years (95% CI 0.24 to 0.68) among women, and 0.23 per 100 person-years (95% CI 0.09 to 0.41) among men (Fig 2).

### HIV incidence among PrEP initiators compared to HIV incidence among recent matched controls prior to PrEP availability

**Fig 3:**
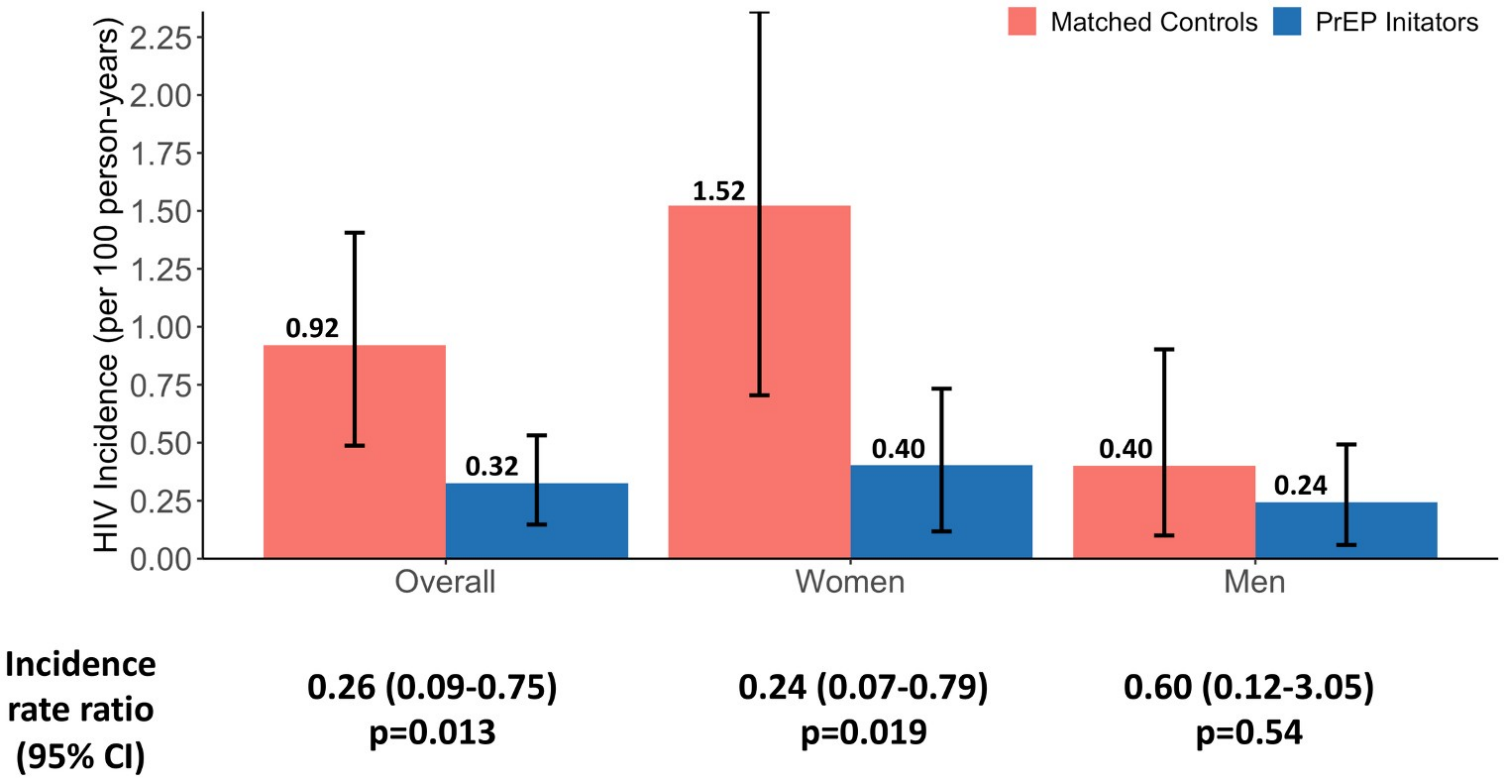
Observed HIV incidence rate among PrEP initiators compared to HIV incidence among recent matched controls prior to PrEP availability, overall and stratified by sex. HIV incidence rate per 100 person-years in 8 study communities with propensity score–matched recent historical controls. CI, confidence interval; PrEP, pre-exposure prophylaxis.

In 8 of the 16 study communities, we compared observed HIV incidence among PrEP initiators to HIV incidence without PrEP, based on propensity score–matched recent controls from the year before PrEP was available. Median follow-up time among matched recent controls was 0.90 years (interquartile range [IQR] 0.83 to 0.97) compared to 1.61 years (IQR 1.02 to 2.45) among PrEP initiators. Among matched recent controls (in the absence of PrEP), there were 17 incident HIV infections over 1,848 person-years of follow-up; the HIV incidence rate was 0.92 per 100 person-years (95% CI 0.49 to 1.41; S5 Table). Among PrEP initiators in the same 8 communities, there were 11 incident HIV infections over 3,393 person-year of follow-up; the HIV incidence rate was 0.32 per 100 person-years (95% CI 0.15 to 0.53), corresponding to 74% lower HIV incidence among PrEP initiators compared to matched recent controls (adjusted incidence rate ratio [aIRR] 0.26, 95% CI 0.09 to 0.75; *p* = 0.013; Fig 3). Among women, HIV incidence among matched controls was 1.52 per 100 person-years (95% CI 0.70 to 2.36) compared to 0.40 per 100 person-years (95% CI 0.12 to 0.73) observed among PrEP initiators, corresponding to 76% lower HIV incidence among PrEP initiators compared to matched recent controls (aIRR 0.24, 95% CI 0.07 to 0.79; *p* = 0.019). Among men, HIV incidence among matched controls was 0.40 per 100 person-years (95% CI 0.10 to 0.90) compared to 0.24 per 100 person-years (95% CI 0.06 to 0.49) observed among PrEP initiators, corresponding to 40% lower HIV incidence that was not statistically significant among PrEP initiators compared to matched recent controls (aIRR 0.60, 95% CI 0.12 to 3.05; *p* = 0.54).

Excluding 3 PrEP participants (all women) who seroconverted at the week 4 visit (and were thus possibly acutely infected at enrollment), HIV incidence was 0.31 per 100 person-years (95% CI 0.18 to 0.45) overall and 0.37 per 100 person-years (95% CI 0.19 to 0.59) among women. Observed incidence was 78% lower among PrEP initiators compared to matched recent controls (overall aIRR 0.22, 95% CI 0.07 to 0.67, *p* = 0.0078; among women: aIRR 0.17, 95% CI 0.05 to 0.59, *p* = 0.0054). Stored plasma was not collected at enrollment to determine whether these individuals were acutely infected at the time of PrEP initiation.

### Demographics and adherence levels among participants with incident HIV infection

Among the 25 individuals with incident HIV infection following PrEP initiation, 17/25 (68%) were women (Table 2). Among the 17 women with incident HIV infection, 8 (47%) had a serodifferent partner at PrEP enrollment compared to 1 (13%) of 8 men. At the seroconversion visit, the median age among women was 27 years (range 20 to 62) and among men was 35 years (range 22 to 49). Regarding adherence, 18/25 (72%) participants reported not taking PrEP for >30 days before the seroconversion visit. One participant with incident HIV infection received TDF/3TC as PrEP once (of 10 fills); the remaining participants with incident HIV infection received only TDF/FTC.

**Table 2:** Baseline characteristics of participants with incident HIV infection after PrEP initiation, overall and stratified by sex.

		Overall (*n* = 25)	Women (*n* = 17)	Men (*n* = 8)
Age, years	15–24	7 (28.0%)	5 (29.4%)	2 (25.0%)
	25–34	11 (44.0%)	8 (47.1%)	3 (37.5%)
	35–44	3 (12.0%)	1 (5.9%)	2 (25.0%)
	45–54	2 (8.0%)	1 (5.9%)	1 (12.5%)
	≥55	2 (8.0%)	2 (11.8%)	0 (0.0%)
Educational attainment^a^	Less than primary level	1 (4.0%)	1 (5.9%)	0 (0.0%)
	Primary school level	17 (68.0%)	12 (70.6%)	5 (62.5%)
	Any secondary school level or higher	3 (12.0%)	1 (17.6%)	2 (25.0%)
Occupation^b^	Farmer	8 (32.0%)	7 (41.2%)	1 (12.5%)
	Student	0 (0.0%)	0 (0.0%)	0 (0.0%)
	Fishing, bar, or transportation	6 (24.0%)	1 (5.9%)	5 (62.5%)
	Other informal sector	6 (24.0%)	5 (29.4%)	1 (12.5%)
	Other formal sector	0 (0.0%)	0 (0.0%)	0 (0.0%)
	Unemployed or disabled	1 (4.0%)	1 (5.9%)	0 (0.0%)
	Other or unknown	0 (0.0%)	0 (0.0%)	0 (0.0%)
Marital status^c^	Not married	2 (8.0%)	1 (5.9%)	1 (12.5%)
	Married (monogamous)	8 (32.0%)	4 (23.5%)	4 (50.0%)
	Married (polygamous)	7 (28.0%)	5 (29.4%)	2 (25.0%)
	Divorced, separated, or widowed	4 (16.0%)	4 (23.5%)	0 (0.0%)
Serodifferent partner	Yes	9 (36.0%)	8 (47.1%)	1 (12.5%)
	No or unknown	16 (64.0%)	9 (52.9%)	7 (87.5%)
Circumcision^d^	Medical	NA	NA	2 (25.0%)
	Traditional	NA	NA	1 (12.5%)
	Uncircumcised	NA	NA	4 (50.0%)
Alcohol use^e^	None	18 (72.0%)	14 (82.4%)	4 (50.0%)
	1–7 days per month	0 (0.0%)	0 (0.0%)	0 (0.0%)
	>7 days per month	3 (12.0%)	0 (0.0%)	3 (37.5%)
Mobility^f^	Yes	0 (0.0%)	0 (0.0%)	0 (0.0%)
	No	21 (84.0%)	14 (82.4%)	4 (50.0%)
Region	Western Kenya	10 (40.0%)	6 (35.3%)	4 (50.0%)
	Eastern Uganda	8 (32.0%)	5 (29.4%)	3 (37.5%)
	Western Uganda	7 (28.0%)	6 (35.3%)	1 (12.5%)

^a^Missing data for 1 (12.5%) male and 3 (17.6%) female individuals.

^b^Other formal sector occupations: teaching, government, military, healthcare, and factory work. Other informal sector occupations: shopkeeper, market vendor, hotel worker, homemaker, household worker, miner, and construction. Missing data for 1 (12.5%) male and 3 (17.6%) female individuals.

^c^Missing data for 1 (12.5%) male and 3 (17.6%) female individuals.

^d^Among men. Missing data for 1 (12.5%) individual.

^e^Missing data for 1 (12.5%) male and 3 (17.6%) female individuals.

^f^Mobility defined as migration out of the community for at least 1 month or moved residence within the past 12 months. Missing for 1 (12.5%) male and 3 (17.6%) female individuals.

PrEP, pre-exposure prophylaxis.

Of the 7/25 (28%) participants who reported taking at least 1 dose of PrEP in the last 30 days, 4 reported intermittent PrEP adherence in the last 3 months (2 had tenofovir levels in hair consistent with taking an average of 4 to 6 PrEP doses/week in the last 4 weeks; 1 had levels consistent with 7 doses/week; and 1 had no hair sample available). One participant had 2-class antiretroviral (ARV) drug resistance (described below) and high adherence (tenofovir levels in hair consistent with 7 doses/week). Three participants seroconverted at the week 4 visit and were possibly acutely infected at enrollment; one reported nonadherence to PrEP, while 2 reported adherence (confirmed by hair levels consistent with 7 doses/week).

### Clinical and virologic outcomes among participants with incident HIV infection

Median plasma HIV RNA concentration at the seroconversion visit was 5,871 c/ml (range <40 to 177,293 c/ml) among the 7 participants reporting adherence to PrEP in the last 30 days. Among the 18 participants who reported no recent PrEP use, median HIV RNA was 24,217 c/ml (range 76 to 1.47 million c/ml). Two participants who seroconverted at the week 4 visit and had evidence of high adherence based on tenofovir concentrations in hair had HIV RNA <40 c/ml (with confirmation of seroconversion by western blot or Geenius).

Among the 25 participants with incident HIV infection, 24/25 (96%) started ART, of whom 16/24 (67%) started same day and 21/24 (88%) within 7 days of the seroconversion visit. All 24 participants started standard first-line ART regimens recommended in national guidelines (22 started EFV-based ART; 2 started dolutegravir-based regimens). Of the 22 participants who started ART and were due for repeat HIV RNA per national guidelines, 19 had subsequent HIV RNA measurement and 3 could not be located for repeat testing. One participant had HIV RNA 79,695 c/ml and was found to have drug resistance on stored plasma from the seroconversion visit, described below. Among the 18 others, the HIV RNA concentrations (grouped according to the assay’s limit of detection) were as follows: 3 had HIV RNA <20 c/ml; 2 had <25 c/ml; 4 had <40 c/ml; 1 had 48 c/ml; 1 had <75 c/ml; 1 had 96 c/ml; and 6 had <1,000 c/ml. Overall, 18/19 (95%) had HIV RNA <1,000 c/ml.

### Drug resistance among participants with incident HIV infection

Ten participants who seroconverted had HIV genotyping results from plasma collected at the seroconversion visit. Fifteen participants did not have genotyping results available due to assay failure (*n* = 7), plasma HIV RNA <500 c/ml (*n* = 5), lack of stored plasma sample from the seroconversion visit (*n* = 2), or declining resistance testing (*n* = 1). Two of the 10 participants who underwent genotyping had evidence of drug resistance, while 8 had no viral resistance mutations detected. One participant had mutations in reverse transcriptase (K103N, E138A) likely due to transmitted drug resistance to non-nucleoside reverse transcriptase inhibitors (NNRTI) not related to PrEP use.

The other participant with drug resistance had 2-class ARV resistance, including transmitted NNRTI mutations (K103N, P225H), likely transmitted nucleoside/nucleotide reverse transcriptase inhibitor (NRTI) mutations (D67N, K70R, K219Q; thymidine analogue mutations, conferring low-level TDF resistance), and an M184V mutation (conferring high-level FTC and 3TC resistance) that was either transmitted or acquired on PrEP. This participant had periods of high levels of PrEP adherence based on tenofovir levels consistent with daily dosing separated by missed visits (S4 Fig). HIV RNA at the seroconversion visit was 177,293 c/ml. This participant experienced viral non-suppression (HIV RNA 79,695 c/ml) on TDF/3TC/EFV prior to receipt of the results of drug resistance testing from the seroconversion visit and was subsequently switched to second-line ART.

## Discussion

During population-level HIV testing of over 75,000 individuals in 3 regions across rural Kenya and Uganda, we offered universal PrEP access with flexible service delivery to persons at elevated risk of HIV acquisition. Of more than 15,000 individuals assessed to be at elevated HIV risk, over 5,400 participants started PrEP, and of these, 79% engaged in the PrEP program for follow-up visits. Over 7,150 person-years of follow-up, HIV incidence was 0.35 per 100 person-years among PrEP initiators. In 8 communities with propensity score–matched controls, HIV incidence was 74% lower among PrEP initiators compared to recent controls in the year before PrEP availability. These results provide supporting evidence that, in generalized epidemic settings, universal access to PrEP with flexible service delivery could reduce HIV incidence on top of universal test and treat (UTT) strategies [26]. Moreover, despite concerns from early placebo-controlled PrEP trials among women in which adherence to study product was too low for effectiveness [27,28], our study found lower HIV incidence among women who started open-label PrEP in real-world settings as compared to recent controls.

Our study offered PrEP in the context of universal HIV testing with supported linkage to either treatment or prevention based on HIV status [29]. We used an inclusive approach to define PrEP eligibility for HIV-uninfected individuals (based on serodifferent partnership, a risk score, or self-identified HIV risk). We offered same-day PrEP start (on-site at health fairs), an approach that was generally safe, as our group and others have reported [12,30,31]. We also offered a flexible PrEP delivery model with options for clinic- or community-based follow-up visits for HIV testing and PrEP refills. We posited that this lower-barrier delivery model would allow individuals to engage in taking PrEP who might not otherwise access health systems or facility-based HIV prevention services. Within this flexible model, we found that self-reported adherence was much higher among individuals reporting current HIV risk at follow-up visits compared to PrEP participants overall. As previously reported, among participants who self-reported PrEP adherence and current HIV risk, 66% had tenofovir concentrations in hair reflecting ≥4 PrEP doses per week and 44% had levels reflecting 7 doses/week [12]. Moreover, although many participants stopped PrEP at some point, ongoing program engagement provided opportunities for repeat HIV testing and restarting PrEP at each visit. Indeed, half of those who stopped PrEP later restarted taking it, suggesting that some participants may have been attempting to match PrEP use to periods of risk [32]. As PrEP programs extend eligibility criteria beyond specific risk groups to persons at substantial risk in the general population [21] and expand services outside of health facilities [33] (e.g., at drop-in centers [34]), our model and other low-barrier approaches to PrEP delivery could be adapted and scaled up.

We observed a lower HIV incidence among PrEP initiators as compared with historical controls prior to PrEP availability in the study communities. To our knowledge, our study is the first in sub-Saharan Africa to assess HIV incidence after population-level offer of PrEP, including to individuals outside of specific risk groups. Our results add to evidence from studies in serodifferent couples and younger women that access to PrEP was associated with lower than expected HIV incidence. In a demonstration study that offered both PrEP and ART to serodifferent couples in Kenya and Uganda from 2012 to 2015, HIV incidence was 0.2%, representing a 96% reduction compared to matched historical controls without PrEP [7]. The historical comparator group in the study dated up to 7 years prior and lacked access to ART (per guidelines at the time); thus, the reduction in incidence was due to both ART and PrEP. In another PrEP study that enrolled young women in urban southern Africa [8], HIV incidence was 1.0% compared to an expected incidence of 3.7% based on mathematical modeling [35]. Most recently, in a contraceptive trial among women that added PrEP to the current standard of HIV prevention (including counseling, condoms, and management of sexually transmitted infections) in South Africa, HIV incidence was approximately 50% lower overall (not only among PrEP initiators) after PrEP availability compared to before [36]. Our findings extend the evidence base on the effectiveness of open-label PrEP in sub-Saharan Africa and, to our knowledge, are among the first to demonstrate that offering PrEP at scale can have a substantial impact on HIV incidence.

Among women who initiated PrEP in our study, we found significantly lower HIV incidence compared to matched recent controls. Women remain a critical population for HIV prevention and are disproportionately impacted by HIV both in our study communities and across much of sub-Saharan Africa [1]. Indeed, although half of PrEP initiators in our study were women, they accounted for two-thirds of new infections. Recent population-based studies, including SEARCH, have shown greater reductions in incidence among men than women in the context of scale-up of ART [11,37–39]. Thus, primary prevention approaches, such as PrEP, may be important for reducing incidence among women. Questions have persisted about the effectiveness of PrEP in women in part because in 2 placebo-controlled PrEP trials that enrolled younger women not in stable serodifferent partnerships, adherence was too low to demonstrate efficacy [27,28]. However, PrEP was effective among women in mutually disclosed serodifferent partnerships in a placebo-controlled trial [2] and an open-label demonstration project [7] in East Africa, as well as among heterosexual men and women in a placebo-controlled trial in Botswana [40]. Our results add to the evidence that oral PrEP can lower HIV incidence among women in sub-Saharan Africa, including some of the first evidence among women who are not in serodifferent partnerships.

Our study also provides insights into the impact of PrEP on HIV incidence among men. We found that HIV incidence was 40% lower among men who initiated PrEP compared to historical controls, although this result did not reach statistical significance, possibly due to the small number of new infections in men. Although men have not been systematically prioritized for PrEP in generalized epidemic settings (apart from men in serodifferent couples or MSM), we found substantial interest in PrEP among men, who comprised half of PrEP initiators. One-third of new HIV infections in sub-Saharan Africa occur among men, and primary prevention options for this group, including male circumcision and PrEP, are critically needed for men’s own health [41]. In addition, men are an important link in transmission networks [42,43], and expanding their access to PrEP could accelerate declines in incidence.

The UTT trials demonstrated reductions in HIV incidence when universal HIV testing was combined with robust linkage and access to ART care [26,37,38]. These studies further showed that despite achieving high population-level viral suppression, UTT is not sufficient to reach HIV elimination targets. Our study provides evidence of the added impact of PrEP in communities that had already exceeded the UNAIDS 90-90-90 targets for HIV testing, ART coverage, and viral suppression following UTT. For countries that adopt a UTT approach in areas such as regional hotspots, leveraging the opportunity for HIV prevention, with inclusive eligibility and low-barrier delivery, is supported by our results.

Although we observed lower incidence among PrEP initiators compared to recent controls, PrEP uptake and engagement were lower in youth and mobile individuals, as we have previously described [12]. Thus, additional innovations are needed to reach these critical groups and provide tailored service delivery and adherence support for oral PrEP. Moreover, as on-demand or long-acting prevention modalities (such as the dapivirine vaginal ring [44,45] and injectable cabotegravir [46]) become available, expanding options for prevention may help to achieve further reductions in incidence.

Among the 25 participants who seroconverted after initiating PrEP, rapid ART start was acceptable and feasible and most had excellent virologic outcomes. Earlier PrEP trials in sub-Saharan Africa offered ART start based on CD4 threshold (per guidelines at the time); thus, prior studies characterizing seroconversions on PrEP have largely reported on HIV outcomes in the absence of ART [47–49]. Moreover, few studies have offered rapid ART start (now the standard of care for new HIV diagnoses [50]) after seroconversion on PrEP, and little is known about virologic outcomes in this group. In our study, we offered same-day ART start at the time of positive antibody testing and found that nearly 90% of participants started ART ≤7 days after the seroconversion visit. Among those with HIV RNA testing after ART initiation, 95% achieved HIV RNA <1,000 c/ml with standard first-line ART regimens. At PrEP follow-up visits, our study employed quarterly HIV testing using country-standard serial antibody-based algorithms. This approach may result in delayed detection of acute HIV infection, potentially increasing the risk of acquiring drug resistance while continuing PrEP and impacting virologic response to ART. To our knowledge, our study is among the first to report on virologic outcomes after ART start following seroconversion on oral PrEP in any setting globally [51]. While it is reassuring that most participants who seroconverted in our study achieved viral suppression on standard first-line ART regimens, robust HIV viral load monitoring (including rapid and, increasingly, point-of-care technologies [52]) and access to resistance testing are needed as PrEP use expands.

The only participant who did not achieve viral suppression on repeat testing after ART start was found to have 2-class drug resistance on testing of stored plasma from the seroconversion visit. This participant had evidence of transmitted drug resistance based on NNRTI mutations and NRTI thymidine analogue mutations (which confer low-level TDF resistance and are not commonly selected by TDF/FTC alone), as well as high-level FTC and 3TC resistance (M184V) that was either transmitted or acquired on PrEP. Based on objective adherence data, this participant had evidence of intermittent high-level adherence to PrEP (tenofovir concentrations in hair consistent with daily dosing at multiple visits, including at the seroconversion visit), separated by missed visits (including the 2 visits prior to seroconversion). There are several possible reasons for seroconversion with drug resistance in this case. First, the participant may have been nonadherent to PrEP during periods of risk, leading to seroconversion, followed by adherence to 2-drug PrEP after seroconversion, leading to acquisition of M184V on top of transmitted NRTI and NNRTI resistance. Second, the participant could have been nonadherent to PrEP and become infected with a multi-class drug resistant virus (with all mutations transmitted, including the M184V mutation). Third, if periods of adherence matched periods of risk, seroconversion could have been due to a breakthrough infection in the setting of high-level FTC resistance and low-level TDF resistance, rendering PrEP ineffective [53]. We do not have HIV genotyping data from the likely transmission partner to determine if drug resistance was transmitted or acquired in this case.

HIV acquisition in the setting of high levels of PrEP adherence has been described in several individuals previously; all were men from North America, Europe, or Asia [54–59]. Notably, several of these cases had evidence of resistance only to FTC (M184V) without mutations conferring TDF resistance, suggesting that PrEP breakthrough can occur with FTC resistance alone [54]. For the participant in our study, although breakthrough infection in the setting of high adherence is one possible explanation for seroconversion (and, to our knowledge, would be the first such case described in a woman), as noted above, infection also could have been due to nonadherence during periods of risk. As PrEP is scaled up globally, even with very high effectiveness, we are likely to observe more seroconversions among PrEP users. Further work is needed to systematically characterize reasons for seroconversion on PrEP through the use of pharmacologic adherence metrics (such as measuring PrEP drug levels in hair [60], dried blood spots [61], or plasma [62]) and drug resistance testing.

This study has several strengths. To our knowledge, our study is among the first and largest to assess HIV incidence after offering PrEP at a population level. Over three quarters of PrEP initiators were seen following PrEP initiation, providing among the most complete and longest follow-up (over 7,000 person-years) to date of any open-label PrEP study in sub-Saharan Africa. In addition, our estimate of HIV incidence among recent controls was based on robust population-level HIV testing data in 8 of the 16 study communities in the year prior to PrEP availability. We offered broad access to PrEP using a community-wide HIV testing model previously shown to be similar in cost to other mobile testing approaches [63] and feasible for implementation by community leaders [64].

This study also has limitations. First, we did not compare HIV incidence to a contemporaneous control where PrEP was unavailable. HIV incidence could have been declining in the study communities due to secular trends. Over the 3 years prior to PrEP availability in the study communities, high rates of population-level viral suppression (79%) were achieved, and population-level HIV incidence declined by 0.12 per 100 person-years [11]. Assuming that this trend continued during the PrEP study period, we would expect HIV incidence among matched controls to reduce from 0.92 per 100 person-years (observed 1 year prior to PrEP) to 0.85 per 100 person-years if their follow-up had been contemporaneous. Comparing HIV incidence among PrEP initiators (0.32 per 100 person-years) to this trend-adjusted incidence for matched controls (0.85 per 100 person-years) suggests that HIV incidence would have been 62% lower among PrEP initiators compared to matched contemporaneous controls. Second, our analyses of incidence relied on data from individuals with at least 2 HIV tests. However, repeat testing coverage was >75% among PrEP initiators and >80% among historical controls [11], and there were no notable differences between persons with and without a repeat test (S3 Table). Third, despite propensity score matching and adjustment for known risk groups, it is possible that the risk profiles of PrEP initiators with follow-up testing differed from the selected controls. If individuals using PrEP were at higher risk than their matched counterparts, the estimated change in HIV incidence would be biased toward the null. Finally, because we used antibody-based screening and did not collect plasma samples at PrEP enrollment, we are unable to determine whether 3 individuals who tested positive at the week 4 visit were acutely infected at the time of PrEP initiation. In sensitivity analyses removing these 3 individuals (all women), observed HIV incidence among PrEP initiators and changes in incidence compared to matched controls were similar to the primary results, overall and among women. A limitation of this sensitivity analysis is that the control group did not have repeat testing at week 4, and it is possible that individuals in the control group also had undetected acute infection at the time of baseline testing.

Community-wide HIV testing and universal access to PrEP were associated with lower HIV incidence among persons at elevated HIV risk who initiated PrEP in rural Kenya and Uganda compared to recent controls. Our findings included lower than expected HIV incidence after PrEP initiation among women, for whom declines in new infections have lagged behind men in recent prevention studies without PrEP. Our results suggest that universal access to HIV testing, treatment, and prevention, including rapid provision of PrEP with flexible service delivery, is a promising approach to reduce HIV incidence in generalized epidemic settings. Moving forward, combination approaches to prevention that include comprehensive HIV testing with linkage to oral PrEP, male circumcision, and ultimately, on-demand and long-acting modalities hold promise to further accelerate declines in HIV incidence globally.

## Supporting information

S1 STROBE Checklist (PDF)

S1 Statistical Analysis Plan (PDF)

S1 Fig PrEP program engagement, refills, and self-reported adherence among PrEP initiators, overall and by self-assessed current HIV risk through week 60. Program engagement defined as attendance at a PrEP follow-up visit during scheduled visit weeks. Excludes participants withdrawn or deceased before visit. Self-reported adherence: at least 1 PrEP dose taken in last 3 days. Self-assessed current HIV risk evaluated at each visit among participants engaged in the PrEP program. PrEP, pre-exposure prophylaxis.
(PDF)

S2 Fig PrEP program engagement, refills, and self-reported adherence among PrEP initiators through week 60, by sex. Program engagement defined as attendance at a PrEP follow-up visit during scheduled visit weeks. Excludes participants withdrawn or deceased before visit. Self-reported adherence: at least 1 PrEP dose taken in last 3 days. PrEP, pre-exposure prophylaxis.
(PDF)

S3 Fig PrEP program engagement, refills, and self-reported adherence at week 24 by age–sex strata. Program engagement defined as attendance at a PrEP follow-up visit during scheduled visit weeks. Excludes participants withdrawn or deceased before visit. Self-reported adherence: at least 1 PrEP dose taken in last 3 days. Self-assessed current HIV risk evaluated at each visit among participants engaged in the PrEP program. PrEP, pre-exposure prophylaxis.
(PDF)

S4 Fig Timeline of PrEP follow-up, HIV testing, and adherence to PrEP estimated from tenofovir concentrations in hair for a participant with incident HIV infection with 2-class antiretroviral drug resistance. Doses of PrEP taken per week in the 4 weeks before study visit, estimated based on tenofovir concentrations in hair. Timeline indicates study visit week with days since PrEP initiation in parentheses below visit week for attended visits. Ab, antibody. ART, antiretroviral therapy; PrEP, pre-exposure prophylaxis.
(PDF)

S1 Table Community-specific start dates of baseline and repeat population-level HIV testing for matched controls in 8 study communities. (DOCX)

S2 Table Baseline characteristics of individuals who tested negative for HIV, those assessed to be at elevated HIV risk, and PrEP initiators in 16 communities in rural Kenya and Uganda. PrEP, pre-exposure prophylaxis.
(DOCX)

S3 Table Baseline characteristics of women and men who initiated PrEP in 16 communities in rural Kenya and Uganda. PrEP, pre-exposure prophylaxis.
(DOCX)

S4 Table Baseline characteristics of individuals who initiated PrEP and those with follow-up HIV testing after PrEP initiation. PrEP, pre-exposure prophylaxis.
(DOCX)

S5 Table Number of incident HIV infections, person-time at risk (in years), and HIV incidence rate (per 100 person-years) for PrEP initiators in all 16 study communities, PrEP initiators living in 8 communities where population-based HIV testing was conducted in the year prior to PrEP availability, and matched controls—overall and by sex. PrEP, pre-exposure prophylaxis.
(DOCX)

## Abbreviations

3TC: lamivudine
aIRR: adjusted incidence rate ratio
ART: antiretroviral therapy
ARV: antiretroviral
CI: confidence interval
EFV: efavirenz
IQR: interquartile range
LC-MS/MS: liquid chromatography-tandem mass spectrometry
MSM: men who have sex with men
NNRTI: non-nucleoside reverse transcriptase inhibitor
NRTI: nucleoside/nucleotide reverse transcriptase inhibitor
PrEP: pre-exposure prophylaxis
SEARCH: Sustainable East Africa Research in Community Health
TDF/FTC: tenofovir disoproxil fumarate/emtricitabine
UCSF: University of California, San Francisco
UTT: universal test and treat

## References

[1] UNAIDS. Global AIDS update: Seizing the moment: Tacking entrenched inequalities to end epidemics. Geneva, Switzerland; 2020.

[2] Baeten JM, Donnell D, Ndase P, Mugo NR, Campbell JD, Wangisi J, et al Antiretroviral prophylaxis for HIV prevention in heterosexual men and women. N Engl J Med. 2012;367(5):399–410. Epub 2012 Jul 11. 10.1056/NEJMoa1108524 22784037 PMC3770474

[3] Grant RM, Lama JR, Anderson PL, McMahan V, Liu AY, Vargas L, et al Preexposure chemoprophylaxis for HIV prevention in men who have sex with men. N Engl J Med. 2010;363(27):2587–99. Epub 2010 Nov 23. 10.1056/NEJMoa1011205 21091279 PMC3079639

[4] Cohen MS, Chen YQ, McCauley M, Gamble T, Hosseinipour MC, Kumarasamy N, et al Antiretroviral therapy for the prevention of HIV-1 transmission. N Engl J Med. 2016;375(9):830–9. Epub 2016 Jul 18. 10.1056/NEJMoa1600693 27424812 PMC5049503

[5] Buchbinder SP, Havlir DV. Getting to Zero San Francisco: A collective impact approach. J Acquir Immune Defic Syndr. 2019;82(Suppl 3):S176–S82. Epub 2019 Nov 26. 10.1097/QAI.0000000000002200 31764252 PMC6880800

[6] Grulich AE, Guy R, Amin J, Jin F, Selvey C, Holden J, et al Population-level effectiveness of rapid, targeted, high-coverage roll-out of HIV pre-exposure prophylaxis in men who have sex with men: the EPIC-NSW prospective cohort study. Lancet HIV. 2018;5(11):e629–e37. Epub 2018 Oct 22. 10.1016/S2352-3018(18)30215-7 . 30343026

[7] Baeten JM, Heffron R, Kidoguchi L, Mugo NR, Katabira E, Bukusi EA, et al Integrated delivery of antiretroviral treatment and pre-exposure prophylaxis to HIV-1-serodiscordant couples: a prospective implementation study in Kenya and Uganda. PLoS Med. 2016;13(8):e1002099 Epub 2016 Aug. 10.1371/journal.pmed.1002099 27552090 PMC4995047

[8] Celum C, Mgodi N, Bekker LG, Hosek S, Donnell D, Anderson P, et al. editors. PrEP adherence and effect of drug level feedback among young African women in HPTN 082. 10th IAS Conference on HIV Science; 2019; Mexico City, Mexico.

[9] Wahome EW, Graham SM, Thiong’o AN, Mohamed K, Oduor T, Gichuru E, et al PrEP uptake and adherence in relation to HIV-1 incidence among Kenyan men who have sex with men. EClinicalMedicine. 2020;26:100541 Epub 2020 Oct 23. 10.1016/j.eclinm.2020.100541 33089128 PMC7565200

[10] Mboup A, Behanzin L, Guedou FA, Nassirou G, Goma-Matsetse E, Giguere K, et al Early antiretroviral therapy and daily pre-exposure prophylaxis for HIV prevention among female sex workers in Cotonou, Benin: a prospective observational demonstration study. J Int AIDS Soc. 2018;21:e25208 Epub 22 November 2018. 10.1002/jia2.25208 31291057 PMC6287093

[11] Havlir DV, Balzer LB, Charlebois ED, Clark TD, Kwarisiima D, Ayieko J, et al HIV testing and treatment with the use of a community health approach in rural Africa. N Engl J Med. 2019;381(3):219–29. Epub 2019 Jul 18. 10.1056/NEJMoa1809866 31314966 PMC6748325

[12] Koss CA, Charlebois ED, Ayieko J, Kwarisiima D, Kabami J, Balzer LB, et al Uptake, engagement, and adherence to pre-exposure prophylaxis offered after population HIV testing in rural Kenya and Uganda: 72-week interim analysis of observational data from the SEARCH study. Lancet HIV. 2020;7(4):e249–e61. Epub 2020 Feb 23. 10.1016/S2352-3018(19)30433-3 32087152 PMC7208546

[13] Koss CA, Ayieko J, Mwangwa F, Owaraganise A, Kwarisiima D, Balzer LB, et al Early adopters of Human Immunodeficiency Virus preexposure prophylaxis in a population-based combination prevention study in rural Kenya and Uganda. Clin Infect Dis. 2018;67(12):1853–60. Epub 2018 May 10. 10.1093/cid/ciy390 29741594 PMC6260162

[14] Chamie G, Clark TD, Kabami J, Kadede K, Ssemmondo E, Steinfeld R, et al A hybrid mobile approach for population-wide HIV testing in rural east Africa: an observational study. Lancet HIV. 2016;3(3):e111–9. Epub 2016 Jan 26. 10.1016/S2352-3018(15)00251-9 26939734 PMC4780220

[15] Zheng W, Balzer L, van der Laan M, Petersen M, SEARCH Collaboration. Constrained binary classification using ensemble learning: an application to cost-efficient targeted PrEP strategies. Stat Med. 2018;37(2):261–79. Epub 2017 Apr 7. 10.1002/sim.7296 28384841 PMC5701877

[16] Chamie G, Sang N, Kwarisiima D, Kabami J, Bagala I, Atukunda M, et al. Yield of HIV testing and re-engagement of key populations in Uganda and Kenya. Conference on Retroviruses and Opportunistic Infections. Seattle, Washington, USA; 2019.

[17] Ministry of Health National AIDS and STI Control Programme. Guidelines on use of antiretroviral drugs for treating and preventing HIV infection in Kenya 2016. Nairobi, Kenya: NASCOP; 2016.

[18] World Health Organization. WHO implementation tool for pre-exposure prophylaxis (PrEP) of HIV infection. Module 6: Pharmacists. Geneva, Switzerland; 2017.

[19] Chesney MA, Ickovics JR, Chambers DB, Gifford AL, Neidig J, Zwickl B, et al Self-reported adherence to antiretroviral medication among participants in HIV clinical trials: the AACTG adherence instruments. AIDS Care. 2000;12(3):255–66. 10.1080/09540120050042891 . 10928201

[20] Uganda Ministry of Health. Consolidated guidelines for prevention and treatment of HIV in Uganda. 2016.

[21] Ministry of Health National AIDS and STI Control Programme. Guidelines on use of antiretroviral drugs for treating and preventing HIV infection in Kenya 2018. Nairobi, Kenya: NASCOP; 2018.

[22] Liu AY, Yang Q, Huang Y, Bacchetti P, Anderson PL, Jin C, et al Strong relationship between oral dose and tenofovir hair levels in a randomized trial: Hair as a potential adherence measure for pre-exposure prophylaxis (PrEP). PLoS ONE. 2014;9(1):e83736 10.1371/journal.pone.0083736 24421901 PMC3885443

[23] Koss CA, Hosek SG, Bacchetti P, Anderson PL, Liu AY, Horng H, et al Comparison of measures of adherence to Human Immunodeficiency Virus preexposure prophylaxis among adolescent and young men who have sex with men in the United States. Clin Infect Dis. 2018;66(2):213–9. 10.1093/cid/cix755 29020194 PMC5850042

[24] Sekhon JS. Multivariate and propensity score matching software with automated balance optimization: The Matching package for R. J Stat Softw. 2011;42(7):1–52.

[25] van der Laan MJ, Polley EC, Hubbard AE. Super learner. Stat Appl Genet Mol Biol. 2007;6:Article25 Epub 2007 Sep 16. 10.2202/1544-6115.1309 . 17910531

[26] Havlir D, Lockman S, Ayles H, Larmarange J, Chamie G, Gaolathe T, et al What do the Universal Test and Treat trials tell us about the path to HIV epidemic control? J Int AIDS Soc. 2020;23(2):e25455 Epub 2020 Feb 25. 10.1002/jia2.25455 32091179 PMC7038879

[27] Marrazzo JM, Ramjee G, Richardson BA, Gomez K, Mgodi N, Nair G, et al Tenofovir-based preexposure prophylaxis for HIV infection among African women. N Engl J Med. 2015;372(6):509–18. 10.1056/NEJMoa1402269 25651245 PMC4341965

[28] Van Damme L, Corneli A, Ahmed K, Agot K, Lombaard J, Kapiga S, et al Preexposure prophylaxis for HIV infection among African women. N Engl J Med. 2012;367(5):411–22. Epub 2012 Jul 11. 10.1056/NEJMoa1202614 22784040 PMC3687217

[29] Ayieko J, Petersen ML, Charlebois ED, Brown LB, Clark TD, Kwarisiima D, et al A Patient-centered multicomponent strategy for accelerated linkage to care following community-wide HIV testing in rural Uganda and Kenya. J Acquir Immune Defic Syndr. 2019;80(4):414–22. Epub 2019 Feb 27. 10.1097/QAI.0000000000001939 30807481 PMC6410970

[30] Mikati T, Jamison K, Daskalakis DC. Immediate PrEP initiation at New York City sexual health clinics. Conference on Retroviruses and Opportunistic Infections. Seattle, Washington; 2019.

[31] Kamis KF, Marx GE, Scott KA, Gardner EM, Wendel KA, Scott ML, et al Same-Day HIV pre-exposure prophylaxis (PrEP) initiation during drop-in sexually transmitted diseases clinic appointments Is a highly acceptable, feasible, and safe model that engages individuals at risk for HIV into PrEP care. Open Forum Infect Dis. 2019;6(7):ofz310 Epub 2019 Jul 26. 10.1093/ofid/ofz310 31341933 PMC6641790

[32] Haberer JE, Bangsberg DR, Baeten JM, Curran K, Koechlin F, Amico KR, et al Defining success with HIV pre-exposure prophylaxis: a prevention-effective adherence paradigm. AIDS. 2015;29(11):1277–85. 10.1097/QAD.0000000000000647 26103095 PMC4480436

[33] Kagaayi J, Batte J, Nakawooya H, Kigozi B, Nakigozi G, Stromdahl S, et al Uptake and retention on HIV pre-exposure prophylaxis among key and priority populations in South-Central Uganda. J Int AIDS Soc. 2020;23(8):e25588 Epub 2020 Aug 14. 10.1002/jia2.25588 32785976 PMC7421540

[34] Were D, Musau A, Mutegi J, Ongwen P, Manguro G, Kamau M, et al Using a HIV prevention cascade for identifying missed opportunities in PrEP delivery in Kenya: results from a programmatic surveillance study. J Int AIDS Soc. 2020;23(Suppl 3):e25537 Epub 2020 Jul 1. 10.1002/jia2.25537 32602658 PMC7325512

[35] Moore JR, Donnell DJ, Boily MC, Mitchell KM, Delany-Moretlwe S, Bekker LG, et al Model-based predictions of HIV incidence among African women using HIV risk behaviors and community-level data on male HIV prevalence and viral suppression. J Acquir Immune Defic Syndr. 2020;85(4):423–9. Epub 2020 Nov 3. 10.1097/QAI.0000000000002481 33136739 PMC7670079

[36] Donnell D, Beesham I, Welch JD, Heffron R, Pleaner M, Kidoguchi L, et al. Incorporating PrEP into standard of prevention in a clinical trial is associated with reduced HIV incidence: Evidence from the ECHO Trial. 23rd International AIDS Conference (AIDS 2020: Virtual), 2020.

[37] Hayes RJ, Donnell D, Floyd S, Mandla N, Bwalya J, Sabapathy K, et al Effect of universal testing and treatment on HIV incidence—HPTN 071 (PopART). N Engl J Med. 2019;381(3):207–18. Epub 2019 Jul 18. 10.1056/NEJMoa1814556 31314965 PMC6587177

[38] Makhema J, Wirth KE, Pretorius Holme M, Gaolathe T, Mmalane M, Kadima E, et al Universal testing, expanded treatment, and incidence of HIV infection in Botswana. N Engl J Med. 2019;381(3):230–42. Epub 2019 Jul 18. 10.1056/NEJMoa1812281 31314967 PMC6800102

[39] Grabowski MK, Serwadda DM, Gray RH, Nakigozi G, Kigozi G, Kagaayi J, et al HIV prevention efforts and incidence of HIV in Uganda. N Engl J Med. 2017;377(22):2154–66. Epub 2017 Nov 25. 10.1056/NEJMoa1702150 29171817 PMC5627523

[40] Thigpen MC, Kebaabetswe PM, Paxton LA, Smith DK, Rose CE, Segolodi TM, et al Antiretroviral preexposure prophylaxis for heterosexual HIV transmission in Botswana. N Engl J Med. 2012;367(5):423–34. Epub 2012 Jul 11. 10.1056/NEJMoa1110711 . 22784038

[41] Grimsrud A, Ameyan W, Ayieko J, Shewchuk T. Shifting the narrative: from “the missing men” to “we are missing the men”. J Int AIDS Soc. 2020;23(Suppl 2):e25526 Epub 2020 Jun 27. 10.1002/jia2.25526 32589325 PMC7319250

[42] de Oliveira T, Kharsany AB, Graf T, Cawood C, Khanyile D, Grobler A, et al Transmission networks and risk of HIV infection in KwaZulu-Natal, South Africa: a community-wide phylogenetic study. Lancet HIV. 2017;4(1):e41–e50. Epub 2016 Dec 1. 10.1016/S2352-3018(16)30186-2 27914874 PMC5479933

[43] Novitsky V, Zahralban-Steele M, Moyo S, Nkhisang T, Maruapula D, McLane MF, et al Mapping of HIV-1C transmission networks reveals extensive spread of viral lineages across villages in Botswana treatment-as-prevention trial. J Infect Dis. 2020 Epub 2020 Jun 4. 10.1093/infdis/jiaa276 . 32492145 PMC7936922

[44] Baeten JM, Palanee-Phillips T, Brown ER, Schwartz K, Soto-Torres LE, Govender V, et al Use of a vaginal ring containing dapivirine for HIV-1 prevention in women. N Engl J Med 2016;375(22):2121–32. Epub 2016 Feb 22. 10.1056/NEJMoa1506110 26900902 PMC4993693

[45] Nel A, van Niekerk N, Kapiga S, Bekker L, Gama C, Gill K, et al Safety and efficacy of dapivirine vaginal ring for HIV prevention in women. N Engl J Med. 2016;375(22):2133–43. 10.1056/NEJMoa1602046 27959766

[46] Landovitz RJ, Donnell D, Clement M, Hanscom B, Cottle L, Coelho L, et al. HPTN 083 Final Results: Pre-exposure prophylaxis containing long-acting injectable cabotegravir is safe and highly effective for cisgender men and transgender women who have sex with men. 23rd International AIDS Conference (AIDS 2020: Virtual), 2020.

[47] Chirwa LI, Johnson JA, Niska RW, Segolodi TM, Henderson FL, Rose CE, et al CD4(+) cell count, viral load, and drug resistance patterns among heterosexual breakthrough HIV infections in a study of oral preexposure prophylaxis. AIDS. 2014;28(2):223–6. Epub 2013 Dec 24. 10.1097/QAD.0000000000000102 . 24361682

[48] Grant RM, Liegler T, Defechereux P, Kashuba AD, Taylor D, Abdel-Mohsen M, et al Drug resistance and plasma viral RNA level after ineffective use of oral pre-exposure prophylaxis in women. AIDS. 2015;29(3):331–7. Epub 2014 Dec 17. 10.1097/QAD.0000000000000556 . 25503265

[49] Riddler SA, Husnik M, Ramjee G, Premrajh A, Tutshana BO, Pather A, et al HIV disease progression among women following seroconversion during a tenofovir-based HIV prevention trial. PLoS ONE. 2017;12(6):e0178594 Epub 2017 Jun 29. 10.1371/journal.pone.0178594 28658251 PMC5489164

[50] World Health Organization. Guidelines for managing advanced HIV disease and rapid initiation of antiretroviral therapy. Geneva, Switzerland; 2017. 29341560

[51] Tittle V, Boffito M, McOwan A, Whitlock G. Dean Street Collaborative Group. Antiretroviral resistance and management after pre-exposure to prophylaxis. Lancet HIV. 2020;7(2):e84 Epub 2020 Feb 7. 10.1016/S2352-3018(19)30404-7 . 32027853

[52] Drain PK, Dorward J, Violette LR, Quame-Amaglo J, Thomas KK, Samsunder N, et al Point-of-care HIV viral load testing combined with task shifting to improve treatment outcomes (STREAM): findings from an open-label, non-inferiority, randomised controlled trial. Lancet HIV. 2020;7(4):e229–e37. Epub 2020 Feb 28. 10.1016/S2352-3018(19)30402-3 32105625 PMC7183312

[53] Knox DC, Anderson PL, Harrigan PR, Tan DH. Multidrug-resistant HIV-1 infection despite preexposure prophylaxis. N Engl J Med 2017;376(5):501–2. Epub 2017 Feb 2. 10.1056/NEJMc1611639 . 28146652

[54] Cohen SE, Sachdev D, Lee SA, Scheer S, Bacon O, Chen MJ, et al Acquisition of tenofovir-susceptible, emtricitabine-resistant HIV despite high adherence to daily pre-exposure prophylaxis: a case report. Lancet HIV. 2018 Epub 2018 Dec 7. 10.1016/S2352-3018(18)30288-1 30503324 PMC6541554

[55] Colby DJ, Kroon E, Sacdalan C, Gandhi M, Grant RM, Phanuphak P, et al Acquisition of multidrug-resistant Human Immunodeficiency Virus type 1 infection in a patient taking preexposure prophylaxis. Clin Infect Dis. 2018;67(6):962–4. Epub 2018 Jul 3. 10.1093/cid/ciy321 29961859 PMC6117447

[56] Hoornenborg E, Prins M, Achterbergh RCA, Woittiez LR, Cornelissen M, Jurriaans S, et al Acquisition of wild-type HIV-1 infection in a patient on pre-exposure prophylaxis with high intracellular concentrations of tenofovir diphosphate: a case report. Lancet HIV. 2017;4(11):e522–e8. Epub 2017 Sep 19. 10.1016/S2352-3018(17)30132-7 . 28919303

[57] Markowitz M, Grossman H, Anderson PL, Grant R, Gandhi M, Horng H, et al Newly acquired infection with multidrug-resistant HIV-1 in a patient adherent to preexposure prophylaxis. J Acquir Immune Defic Syndr. 2017;76(4):e104–e6. Epub 2017 Oct 28. 10.1097/QAI.0000000000001534 29076941 PMC5792163

[58] Spinelli MA, Lowery B, Shuford JA, Spindler J, Kearney MF, McFarlane JR, et al Use of drug-level testing and single-genome sequencing to unravel a case of HIV seroconversion on PrEP. Clin Infect Dis. 2020 Epub 2020 Jul 21. 10.1093/cid/ciaa1011 . 32686825 PMC8315126

[59] Thaden JT, Gandhi M, Okochi H, Hurt CB, McKellar MS. Seroconversion on preexposure prophylaxis: a case report with segmental hair analysis for timed adherence determination. AIDS. 2018;32(9):F1–F4. Epub 2018 Apr 24. 10.1097/QAD.0000000000001825 29683856 PMC6140333

[60] Gandhi M, Glidden DV, Mayer K, Schechter M, Buchbinder S, Grinsztejn B, et al Association of age, baseline kidney function, and medication exposure with declines in creatinine clearance on pre-exposure prophylaxis: an observational cohort study. Lancet HIV. 2016;3(11):e521–e8. Epub 2016 Aug 31. 10.1016/S2352-3018(16)30153-9 27658870 PMC5085869

[61] Anderson PL, Liu AY, Castillo-Mancilla JR, Gardner EM, Seifert SM, McHugh C, et al Intracellular tenofovir-diphosphate and emtricitabine-triphosphate in dried blood spots following directly observed therapy. Antimicrob Agents Chemother. 2018;62(1). 10.1128/AAC.01710-17 29038282 PMC5740314

[62] Hendrix CW, Andrade A, Bumpus NN, Kashuba AD, Marzinke MA, Moore A, et al Dose frequency ranging pharmacokinetic study of tenofovir-emtricitabine after directly observed dosing in healthy volunteers to establish adherence benchmarks (HPTN 066). AIDS Res Hum Retroviruses. 2016;32(1):32–43. Epub 2015 Oct 15. 10.1089/AID.2015.0182 26414912 PMC4692123

[63] Chang W, Chamie G, Mwai D, Clark TD, Thirumurthy H, Charlebois ED, et al Implementation and Operational Research: Cost and efficiency of a hybrid mobile multidisease testing approach with high HIV testing coverage in East Africa. J Acquir Immune Defic Syndr. 2016;73(3):e39–e45. Epub 2016 Oct 16. 10.1097/QAI.0000000000001141 27741031 PMC5089839

[64] Kabami J, Chamie G, Kwarisiima D, Biira E, Ssebutinde P, Petersen M, et al Evaluating the feasibility and uptake of a community-led HIV testing and multi-disease health campaign in rural Uganda. J Int AIDS Soc. 2017;20(1):21514 Epub 2017 Apr 14. 10.7448/IAS.20.1.21514 28406269 PMC5515014

