# Supplementary material for: HIV incidence after pre-exposure prophylaxis initiation among women and men at elevated HIV risk: A population-based study in rural Kenya and Uganda
Source: PLoS Med. 2021 Feb 9;18(2):e1003492. doi: 10.1371/journal.pmed.1003492 (PMC7872279; doi:10.1371/journal.pmed.1003492)
Supplement: S1 Statistical Analysis Plan — (PDF) [file pmed.1003492.s002.pdf]

Sustainable East Africa Research in Community Health

Statistical Analysis Plan

Project: HIV incidence among PrEP Initiators in SEARCH

Laura B. Balzer, PhD*
Joshua Nugent, MEd*
Catherine A. Koss, MD**
for the SEARCH Collaboration

December 11, 2019
Version 1.0

Table of Contents

1.0 Overview **2**

2.0 Characterize the PrEP cascade **2**

3.0 Incidence rate of HIV among PrEP initiators **3**

4.0 HIV incidence observed compared to expected without PrEP **4**

4.1 Propensity score matching to select controls **4**

4.2 Estimation of the incidence rate ratio **5**

5. Description of participants with incident HIV infection **5**

Table 1: **7**

References **9**

*University of Massachusetts, Amherst
**University of California, San Francisco

1.0 Overview

Sustainable East Africa Research in Community Health (SEARCH) (NCT01864603) is a population-based study in rural Kenya and Uganda. Beginning in June 2016, the study implemented a population-level HIV pre-exposure prophylaxis (PrEP) intervention in 16 communities before PrEP was available through national guidelines. PrEP was introduced in successive communities over one year (2016-2017) during population-based HIV testing (through multi-disease health fairs and home-based testing). PrEP was offered to persons at elevated risk of HIV acquisition, and a flexible service delivery model was provided with PrEP follow-up visits (including HIV testing) at clinics or community-based sites for up to 144 weeks.

In 8 of the 16 study communities, population-based HIV testing was also conducted (through the SEARCH study) one year prior to the start of the PrEP intervention (over 2015-2016, depending on the study community). These 8 communities can therefore provide data on HIV incidence before and after PrEP availability.

The goals of these analyses are to

1. Characterize the PrEP cascade.
2. Estimate the incidence rate of HIV among PrEP initiators.
3. Estimate the incidence rate of HIV that would have occurred for this population without PrEP.
4. Test the null hypothesis that the observed HIV incidence among PrEP initiators was equal to expected incidence without PrEP (adjusted incidence rate ratio=1) – overall and by sex.
5. Describe the demographics, adherence levels, clinical outcomes, virologic outcomes, and drug resistance among participants with incident HIV infection.

The primary outcome is HIV incidence rate (per 100 person-years) among PrEP initiators with at least one subsequent HIV test after PrEP initiation. All analyses will be conducted in *R*.[1]

Given that this study focused on open-label PrEP in real-world settings, there was no formal sample size calculation.

2.0 Characterize the PrEP cascade

We will characterize the PrEP cascade by calculating the following metrics. All analyses will be conducted among participants of the 16 communities where PrEP was offered. All analyses will be restricted to persons aged 15 years or older.

- Elevated risk: among persons testing negative for HIV, the number and proportion assessed to be at elevated risk (in serodifferent partnerships, identified by an empirical HIV risk prediction algorithm,[2] or self-identified HIV risk).
- PrEP uptake: among persons assessed to be at elevated HIV risk, the number and proportion who initiated PrEP (defined as receiving pills from the study from June 2016-November 2019).

- Program engagement: among persons who initiated PrEP, the number and proportion who attended follow-up visits during the following intervals, defined with respect to their enrollment date and with censoring at death or study withdrawal:
  - Week 4 (-14/+28 days)
  - Week 12 (-27/+42 days)
  - Weeks 24 through 144 (-41/+42 days)
- At each follow-up visit, we will calculate
  - Number and proportion self-reporting current HIV risk
  - Number and proportion receiving PrEP medications refills (overall and among those self-reporting current HIV risk)
  - Number and proportion self-reporting PrEP adherence – at least 1 of the past 3 doses taken (overall and among those self-reporting current HIV risk)
- Among all persons who initiated PrEP, we will also calculate the number and proportion who ever engaged in the PrEP program (attended ≥1 follow-up visit), ever received PrEP medication refills (≥1 refill received), or ever self-reported PrEP adherence (at ≥1 follow-up visit), both overall and by sex.
- In age-sex strata (among women and men ages 15-24, 25-34, 35-44, and ≥45 years), we will calculate PrEP program engagement, refills, and self-reported adherence at visit week 24.

Participants not seen at follow-up visits will be assumed to be non-adherent.

We will also provide a Consort diagram, detailing the flow of participants in the study. Descriptive tables of PrEP initiators will be provided overall and by sex.

3.0 Incidence rate of HIV among PrEP initiators

Our target population is persons aged ≥15 years who initiated in PrEP in the 16 study communities in rural Kenya and Uganda. The analytic population consists of persons living in the study communities (regardless of stability or official residency in the community), who started PrEP, are aged ≥15 years, and have at least one HIV test after initiating PrEP. Our primary outcome is the HIV incidence rate: the number of seroconversions (defined as two positive HIV antibody tests with confirmation by HIV RNA, Western Blot, or Geenius) divided by the total person-time-at-risk. Person-time-at-risk begins on the date of HIV-negative test at or immediately prior to PrEP initiation. Person-time-at-risk ends at death, HIV-positive test (date of positive antibody testing), or last available HIV test date prior to database closure (anticipated June 2020). For individuals testing HIV-positive, we will impute the date of seroconversion as the midpoint between the last HIV-negative test date and first HIV-positive test date.

We will obtain 95% confidence intervals for the incidence rate using 2.5% and 97.5% quantiles of the non-parametric bootstrap, sampling with replacement 5000 times. Inference will be confirmed with the jackknife estimator. We will also estimate the incidence rate among PrEP initiators living in the 8 communities with population-based HIV testing in the year prior to PrEP availability. HIV incidence rates will be calculated overall and by sex.

In a sensitivity analysis, we will exclude participants who seroconverted within one month of the PrEP enrollment visit and thereby may have been acutely infected with HIV at the time of PrEP initiation. We will also conduct a sensitivity analysis excluding non-residents.

4.0 HIV incidence observed compared to expected without PrEP

We do not have HIV incidence data from a contemporaneous control without access to PrEP. Therefore, our estimates of HIV incidence without PrEP will be based on data from 8 of the 16 communities where population-based HIV testing occurred in the year prior to the start of the PrEP intervention.

As historical controls, we could use all participants in these 8 communities, who have known HIV status when PrEP became available (i.e. over 2016-2017) and were aged ≥15 and HIV-negative one year earlier (i.e. over 2015-2016). However, individuals initiating PrEP are likely to be at higher risk of HIV acquisition than members of their community in general. In other words, PrEP is intended for use among HIV-negative individuals who are at high risk of infection - not the general HIV-negative population. Therefore, we will select controls by matching on the estimated propensity score (conditional probability of PrEP uptake, given baseline predictors of HIV risk) and estimate the HIV incidence rate ratio among PrEP initiators relative to their matched controls (analogous to average treatment effect among the treated).[3] Given variations in HIV risk by community, we will conduct matching within community. Thereby, our analyses of observed vs. expected incidence will be restricted to the 8 communities with HIV incidence data before and after PrEP became available.

We could assume that the incidence rate observed among matched controls is representative of what the incidence rate would have been without PrEP. However, we further weaken this assumption by adjusting for additional characteristics, which have previously been shown to predictive of HIV risk acquisition in this setting: women aged 15-24 years, individuals with serodifferent partners, alcohol users, widow(er)s, and persons employed in the transportation, bar, or fishing industries.[4]

4.1 Propensity score matching to select controls

For simplicity, let A denote our primary exposure variable for these analyses. Specifically, A equals 1 for individuals who initiate PrEP and equals 0 for controls. Let W denote the set of predictors of HIV acquisition that are potentially imbalanced between exposure groups and measured at/prior to the start of each risk period (**Table 1**). The conditional probability of being exposed, given the predictors (here, the conditional probability of initiating PrEP, given the risk factors) is known as the "propensity score": $\mathbb{P}(A = 1|W)$.

With the following steps, we will select matched controls based on the estimated propensity score:

1. Screen the set of predictors based on univariate correlations with the outcome of HIV seroconversion (p<0.1).
2. Use Super Learner to estimate the propensity score: the conditional probability of initiating PrEP, given the remaining predictors. Super Learner is an ensemble machine

learning algorithm using sample-splitting to build the best weighted combination of a library of candidate prediction algorithms.[5] Here, our library will include LASSO, ridge regression, elastic net, stepwise regression, and the empirical mean. To calculate the optimal weights, we will use 5-fold cross-validation and the negative log-likelihood loss function. Specifically, we will use the *SuperLearner* package.[6]

3. Within community separately, we will implement one-to-one matching with replacement on the estimated propensity score. We will use the *Match* function from the *Matching* package.[7]

We will examine the propensity score distributions before and after matching. We refer to PrEP initiators with the selected controls as the "matched data".

For each exposure group (PrEP initiators or selected controls), we will calculate the HIV incidence rate – overall and by sex. We will use the same methods for estimation and inference as described in Section 3.0. In sensitivity analyses, we will vary the matching algorithm (i.e. matching ratio, handling of ties, and replacement) as well as exclude PrEP initiators who seroconverted at the week 4 PrEP visit following enrollment.

4.2 Estimation of the incidence rate ratio

Using the matched data, we will regress outcome (indicator HIV seroconversion) on the exposure variable (PrEP initiation) using the log-link (i.e. Poisson regression) and the log-person-time-at-risk as offset. To control for remaining differences between the risk profiles of PrEP initiators and the matched controls, we will adjust for previously identified demographic risk factors: women aged 15-24 years, individuals with serodifferent partners, alcohol users, widow(er)s, and persons employed in transportation, bars, or fishing.[4] We will also include fixed effects for communities. Inference will be based on robust (sandwich) standard errors.[8–10] We will test the null hypothesis that the incidence rate among PrEP initiators is equal to the incidence rate among matched controls (aIRR=1) at the α=0.05 significance-level.

In sensitivity analyses, we will drop the fixed effects for communities, drop the adjustment variables, and drop both the fixed effects and adjustment variables (i.e. conduct a crude analysis). Given the expected rarity of the outcome, sex-specific analyses will not be adjusted for demographic risk factors, but will adjust for communities as fixed effects.

5. Description of participants with incident HIV infection

We will characterize PrEP initiators with incident HIV infection through the following metrics:

- Basic demographics, overall and by sex
- Number and proportion with self-reported adherence, defined as taking at least 1 PrEP dose in last 30 days
- Adherence to PrEP (estimated number of doses taken per week based on analyses of tenofovir concentrations in hair) among participants who self-reported adherence (defined as taking at least 1 PrEP dose in the last 30 days)

- Number and proportion who started ART overall and by time from the date of positive HIV antibody testing (i.e. same-day, within 7 days, or longer)
- Median HIV RNA at the time of positive HIV antibody testing – overall and by self-reported adherence
- Viral suppression (<1000 copies/ml) among participants with HIV RNA measurement after ART initiation
- Number of participants with antiretroviral drug resistance mutations among those with HIV genotyping results

Table 1: Complete list of candidate predictors of HIV acquisition and their descriptions, including handling of missing data. All predictors were measured at the time of population-based HIV testing (i.e. 2015-2016 for controls and 2016-2017 for PrEP initiators).

**DEMOGRAPHICS**	
Age	Age (in years) as a continuous variable as well as the following groupings: 25-34, 35-44, 45-54, 55+; reference as 15-24
Sex	Male sex; reference as female sex
Marital status	Single, married, widowed, divorced/separated; reference as missing
Polygamy	In a polygamous marriage, not in a polygamous marriage; reference as unknown
Education	Completed primary school, completed secondary school or more; reference as less than primary or unknown
Occupation strata	Formal sector (teacher, student, government worker, military worker, health worker, or factory worker); Fishing/bar/transport (fishmonger, fisherman, bar owner, bar worker, transport, or tourism); Informal sector (farmer, shopkeeper, market vendor, hotel worker, homemaker, household worker, construction worker, or mining); Jobless; reference as other (including unknown)
Student	Student; reference as otherwise
Transportation	Transportation (truck, taxi, motorcycle, bike, boat) drivers; reference as otherwise
Fishing	Fisherman or fishmonger; reference as otherwise
Bar worker	Bar worker or bar owner; reference as otherwise
Hotel worker	Hotel or restaurant worker; reference as otherwise
Shopkeeper	Shopkeeper or market vendor; reference as otherwise
Alcohol use	Any alcohol use, no alcohol use; reference as unknown
Region	Uganda-West; Kenya; reference as Uganda-East
**MOBILITY**	
In-migrant	Moved into the community in the last 3 years; reference as otherwise
Stable resident	Living <6 months outside community in the last 12 months; reference as otherwise
Mobile resident	Living 1+ month outside the community in the last 12 months; reference as otherwise
Moved residence	Moved (i.e. changed or shifted) residence in the last 12 months; did not move residence in the last 12 months; reference as unknown
Nights away	Nights spent away from home in past month: no or only a few nights; less than half of the month; more than half of the month; most nights; every night; reference as unknown
**HEALTH**	
Health fair attendance	Attended the health fair at start of risk period; reference as otherwise
Contraceptive use	Using contraceptives; not using contraceptives; reference as unknown
Pregnant	Pregnant; not pregnant; reference as male or unknown

Live birth	At least 1 live birth in the past year; 0 live births in the past year; reference as male or unknown
Male circumcision	Traditional male circumcision; medical male circumcision; not circumcised; reference as unknown or female
**PARTNERS**	
Serodifferent	Partner is living with HIV; partner is HIV-noninfected; reference as otherwise
Serodifferent and unsuppressed HIV RNA	Partner is living with HIV and HIV RNA level >500 copies/mL at the time of population-level testing; reference as otherwise
**HOUSEHOLD FACTORS**	
Relation to household	Related to head-of-household; reference as otherwise
Wealth	Quintiles based on a principal component analysis of household wealth survey and calculated at the level of the household; reference as unknown
Adult of the opposite sex who is living with HIV	At least 1 adult of the opposite sex and living with HIV in the household; no adults of the opposite sex and living with HIV in the household; reference as unknown
**INTERACTIONS**	
Young woman	Woman aged 15-24 years; reference as otherwise
Female bar worker	Female bar worker or bar owner; reference as otherwise
High SES man	Man in highest socioeconomic strata; reference as otherwise
Young woman who is pregnant	Woman aged 15-24 years and reporting current pregnancy; reference as otherwise
Young woman with live birth in last year	Woman aged 15-24 years and reporting at least 1 live birth in the past year; reference as otherwise

References

1. R Core Team. *R: A Language and Environment for Statistical Computing*. R Foundation for Statistical Computing; 2019. http://www.R-project.org

2. Zheng W, Balzer L, Laan M van der, Petersen M, the SEARCH Collaboration. Constrained binary classification using ensemble learning: an application to cost-efficient targeted PrEP strategies. *Statistics in Medicine*. 2018;37(2):262-279. doi:10.1002/sim.7296

3. Stuart EA. Matching Methods for Causal Inference: A Review and a Look Forward. *Statist Sci*. 2010;25(1):1-21. doi:10.1214/09-STS313

4. Balzer LB, Havlir DV, Kamya MR, et al. Machine Learning to Identify Persons at High-Risk of Human Immunodeficiency Virus Acquisition in Rural Kenya and Uganda. *Clin Infect Dis*. doi:10.1093/cid/ciz1096

5. van der Laan MJ, Polley EC, Hubbard AE. Super learner. *Statistical Applications in Genetics and Molecular Biology*. 2007;6(1):25. doi:10.2202/1544-6115.1309

6. Polley E, LeDell E, Kennedy C, van der Laan M. *SuperLearner: Super Learner Prediction*.; 2019. Accessed July 15, 2019. http://CRAN.R-project.org/package=SuperLearner

7. Sekhon JS. Multivariate and Propensity Score Matching Software with Automated Balance Optimization: the Matching package for R. *Journal of Statistical Software*. 2011;42(7):1–52.

8. Liang KY, Zeger SL. Longitudinal Data Analysis Using Generalized Linear Models. *Biometrika*. 1986;73(1):13-22.

9. Zeileis A. Econometric Computing with HC and HAC Covariance Matrix Estimators. *Journal of Statistical Software*. 2004;11(1):1-17. doi:10.18637/jss.v011.i10

10. Zeileis A. Object-oriented Computation of Sandwich Estimators. *Journal of Statistical Software*. 2006;16(1):1-16. doi:10.18637/jss.v016.i09
