# Supplementary material for: HIV incidence after pre-exposure prophylaxis initiation among women and men at elevated HIV risk: A population-based study in rural Kenya and Uganda
Source: PLoS Med. 2021 Feb 9;18(2):e1003492. doi: 10.1371/journal.pmed.1003492 (PMC7872279; doi:10.1371/journal.pmed.1003492)
Supplement: S1 Fig — Program engagement defined as attendance at a PrEP follow-up visit during scheduled visit weeks. Excludes participants withdrawn or deceased before visit. Self-reported adherence: at least 1 PrEP dose taken in last 3 days. Self-assessed current HIV risk evaluated at each visit among participants engaged in the PrEP program. PrEP, pre-exposure prophylaxis. (PDF) [file pmed.1003492.s003.pdf]

S1 Fig. PrEP program engagement, refills, self-reported adherence among PrEP initiators, overall and by self-assessed current HIV risk through week 60

A. Overall

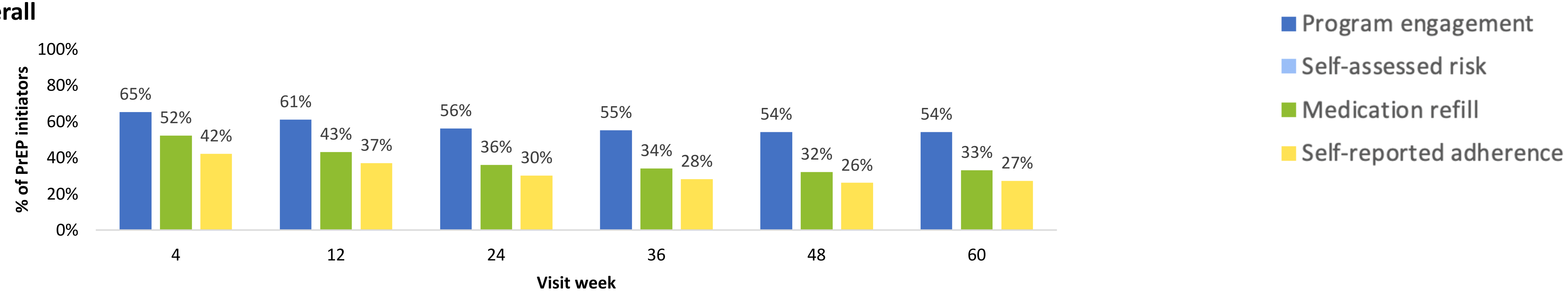

	4	12	24	36	48	60
Eligible for visit	5398	5352	5299	5228	5182	5094

B. Participants reporting self-assessed current HIV risk at follow-up visits, and the proportion receiving refills and self-reporting adherence

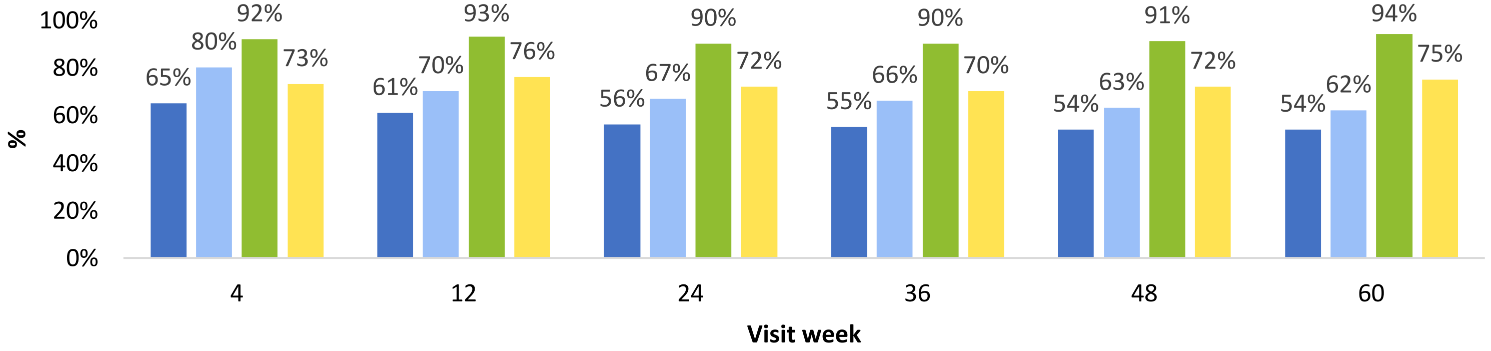

	4	12	24	36	48	60
Program engagement/ PrEP initiators eligible for visit	3512/5398	3286/5352	2987/5299	2893/5228	2784/5182	2758/5094
Self-assessed current risk/ engaged in program	2826/3512	2297/3286	1987/2987	1919/2893	1752/2784	1711/2758
Medication refill/ self-assessed current risk	2605/2826	2130/2297	1785/1987	1735/1919	1600/1752	1601/1711
Adherence/ self-assessed current risk	2053/2826	1747/2297	1428/1987	1349/1919	1267/1752	1277/1711
