# Supplementary material for: HIV incidence after pre-exposure prophylaxis initiation among women and men at elevated HIV risk: A population-based study in rural Kenya and Uganda
Source: PLoS Med. 2021 Feb 9;18(2):e1003492. doi: 10.1371/journal.pmed.1003492 (PMC7872279; doi:10.1371/journal.pmed.1003492)

S2 Fig. PrEP program engagement, refills, self-reported adherence among PrEP initiators through week 60, by sex

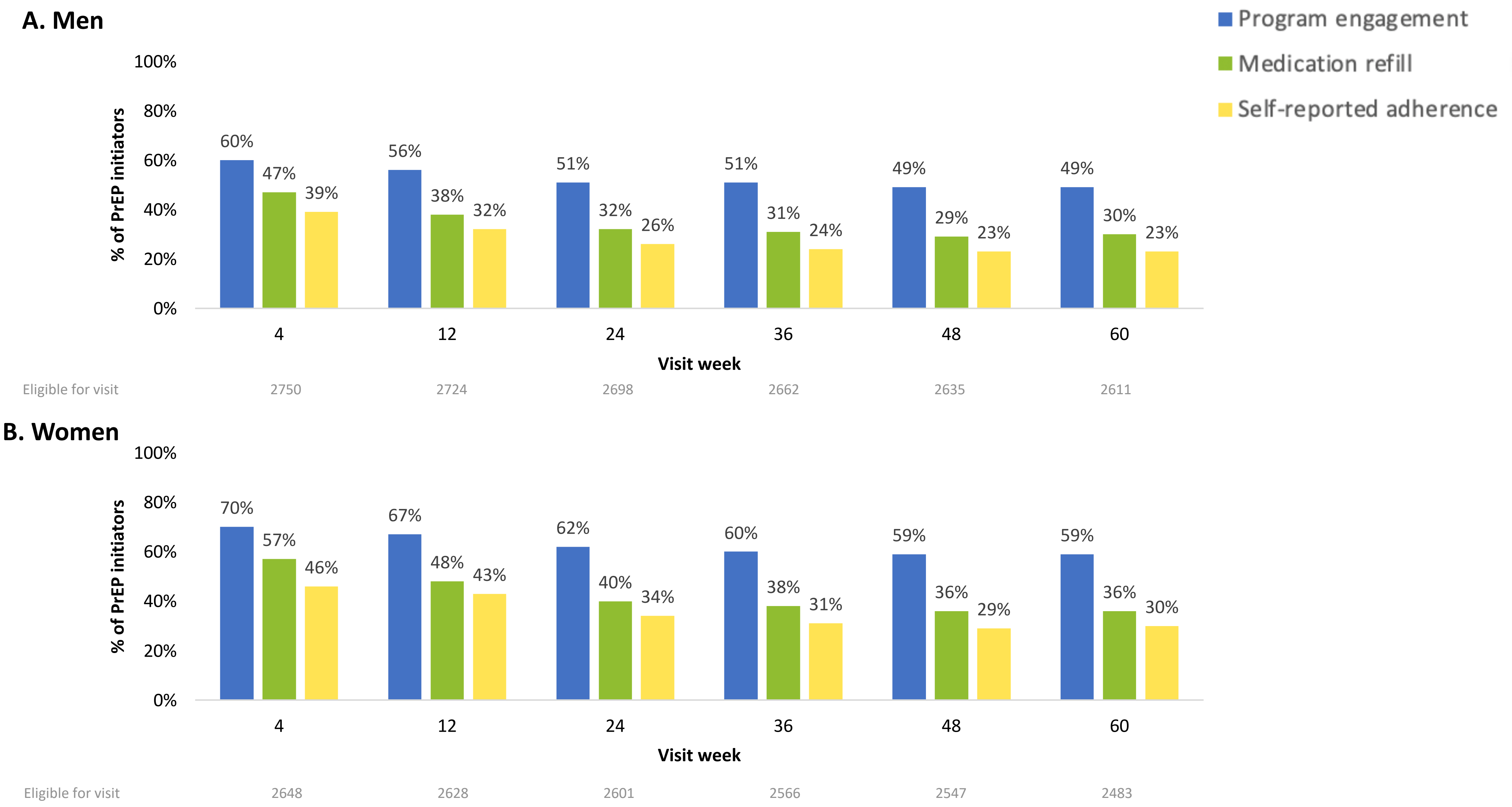

Supplement: S2 Fig — Program engagement defined as attendance at a PrEP follow-up visit during scheduled visit weeks. Excludes participants withdrawn or deceased before visit. Self-reported adherence: at least 1 PrEP dose taken in last 3 days. PrEP, pre-exposure prophylaxis. (PDF) [file pmed.1003492.s004.pdf]
