# Supplementary material for: HIV incidence after pre-exposure prophylaxis initiation among women and men at elevated HIV risk: A population-based study in rural Kenya and Uganda
Source: PLoS Med. 2021 Feb 9;18(2):e1003492. doi: 10.1371/journal.pmed.1003492 (PMC7872279; doi:10.1371/journal.pmed.1003492)
Supplement: S3 Fig — Program engagement defined as attendance at a PrEP follow-up visit during scheduled visit weeks. Excludes participants withdrawn or deceased before visit. Self-reported adherence: at least 1 PrEP dose taken in last 3 days. Self-assessed current HIV risk evaluated at each visit among participants engaged in the PrEP program. PrEP, pre-exposure prophylaxis. (PDF) [file pmed.1003492.s005.pdf]

S3 Fig. PrEP program engagement, refills, self-reported adherence at week 24 by age/sex strata

A. Overall

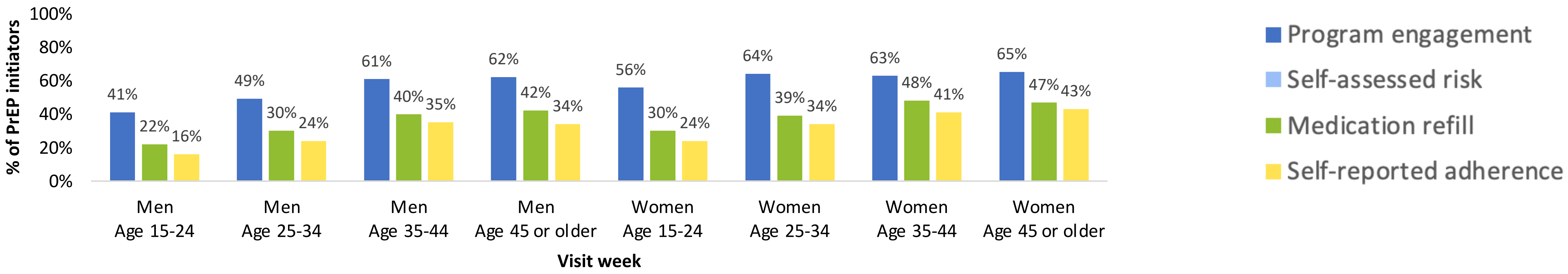

Eligible for visit	812	877	512	497	727	947	582	345

B. Participants reporting self-assessed current HIV risk at follow-up visits, and the proportion receiving refills and self-reporting adherence

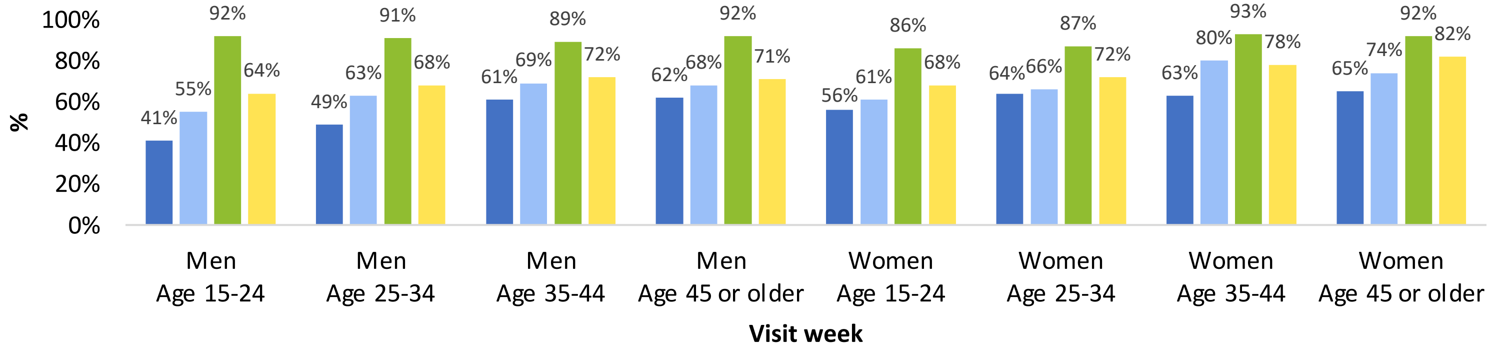

Program engagement/ PrEP initiators eligible for visit	334/812	433/877	310/512	310/497	404/727	604/947	368/582	224/345
Self-assessed current risk/ engaged in program	184/334	274/433	214/310	212/310	245/404	399/604	294/368	165/224
Medication refill/ self-assessed current risk	170/184	248/274	190/214	195/212	210/245	349/399	272/294	151/165
Adherence/ self-assessed current risk	117/184	185/274	155/214	151/212	167/245	288/399	229/294	136/165
