# Supplementary material for: HIV incidence after pre-exposure prophylaxis initiation among women and men at elevated HIV risk: A population-based study in rural Kenya and Uganda
Source: PLoS Med. 2021 Feb 9;18(2):e1003492. doi: 10.1371/journal.pmed.1003492 (PMC7872279; doi:10.1371/journal.pmed.1003492)

S4 Fig. Timeline of PrEP follow-up, HIV testing, and adherence to PrEP estimated from tenofovir concentrations in hair for a participant with incident HIV infection with two-class antiretroviral drug resistance

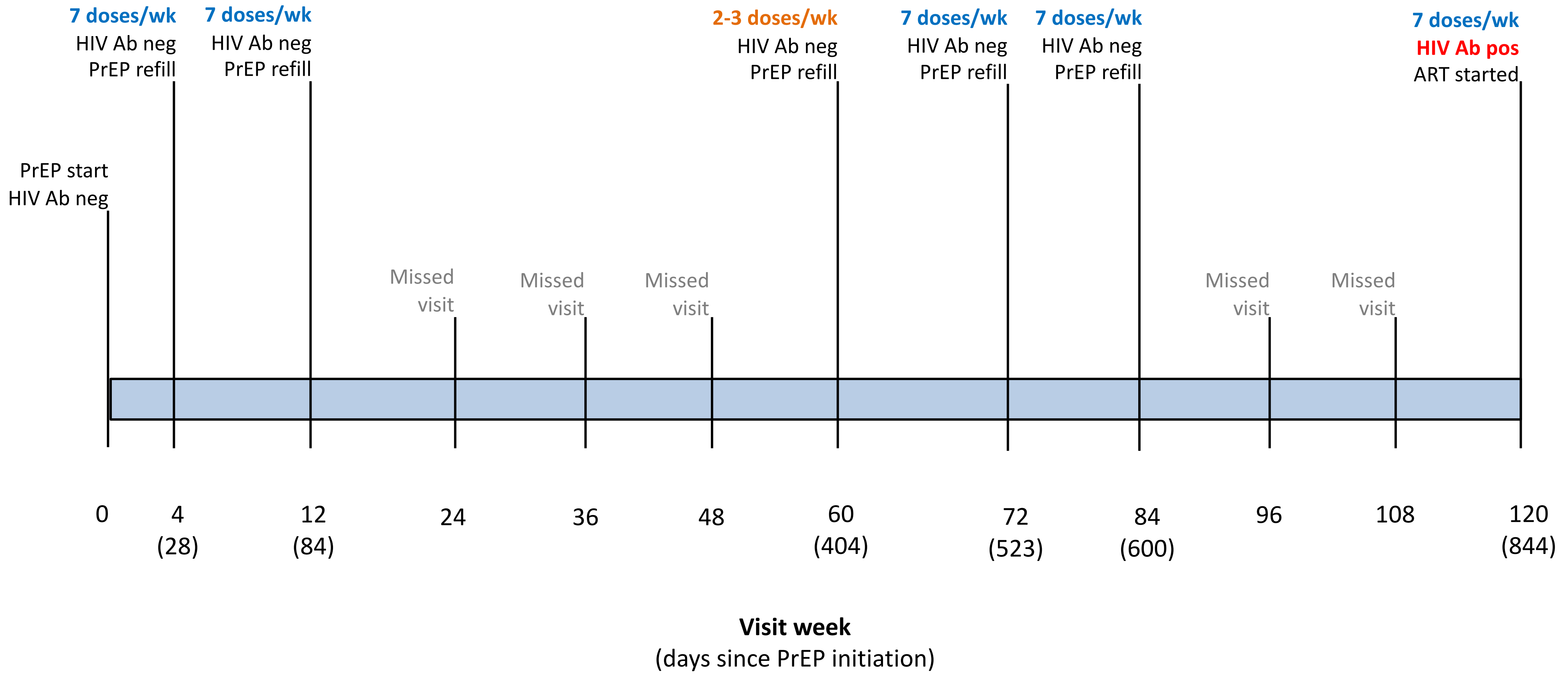

Supplement: S4 Fig — Doses of PrEP taken per week in the 4 weeks before study visit, estimated based on tenofovir concentrations in hair. Timeline indicates study visit week with days since PrEP initiation in parentheses below visit week for attended visits. Ab, antibody. ART, antiretroviral therapy; PrEP, pre-exposure prophylaxis. (PDF) [file pmed.1003492.s006.pdf]
