# Supplementary material for: HIV incidence after pre-exposure prophylaxis initiation among women and men at elevated HIV risk: A population-based study in rural Kenya and Uganda
Source: PLoS Med. 2021 Feb 9;18(2):e1003492. doi: 10.1371/journal.pmed.1003492 (PMC7872279; doi:10.1371/journal.pmed.1003492)
Supplement: S1 Table — (DOCX) [file pmed.1003492.s007.docx]

**S1 Table. Community-specific start dates of baseline and repeat population-level HIV testing for matched controls in 8 study communities**

| **Community name** | **Start of baseline HIV testing for matched controls^a^** | **Start of repeat HIV testing for**  **matched controls^a,b^** |
| --- | --- | --- |
| Bugamba | 8/26/15 | 7/23/16 |
| Nankoma | 9/17/15 | 7/12/16 |
| Ogongo | 11/9/15 | 8/30/16 |
| Muyembe | 1/12/16 | 11/29/16 |
| Sena | 3/8/16 | 1/31/17 |
| Ruhoko | 3/11/16 | 3/2/17 |
| Kameke | 5/3/16 | 4/25/17 |
| Sibuoche | 5/16/16 | 5/2/17 |

1. Dates listed in month/day/year format. Communities listed in chronological order based on start of baseline HIV testing.
2. Start of repeat HIV testing coincides with the start of PrEP intervention.
