# Supplementary material for: HIV incidence after pre-exposure prophylaxis initiation among women and men at elevated HIV risk: A population-based study in rural Kenya and Uganda
Source: PLoS Med. 2021 Feb 9;18(2):e1003492. doi: 10.1371/journal.pmed.1003492 (PMC7872279; doi:10.1371/journal.pmed.1003492)
Supplement: S2 Table — PrEP, pre-exposure prophylaxis. (DOCX) [file pmed.1003492.s008.docx]

**S2 Table. Baseline characteristics of individuals who tested negative for HIV, those assessed to be at elevated HIV risk, and PrEP initiators in 16 communities in rural Kenya and Uganda**

|  |  | **Tested negative**  **for HIV**  **(N = 74,541)** | **Elevated HIV risk**  **(N = 15,632)** | **PrEP initiators**  **(N = 5,447)** |
| --- | --- | --- | --- | --- |
| Sex | Female | 40,401 (54.2%) | 7,627 (48.8%) | 2,674 (49.1%) |
|  | Male | 34,140 (45.8%) | 8,005 (51.2%) | 2,773 (50.9%) |
| Age, years | 15-24 | 27,606 (37.0%) | 5,695 (36.4%) | 1,582 (29.0%) |
|  | 25-34 | 16,322 (21.9%) | 5,570 (35.6%) | 1,879 (34.5%) |
|  | 35-44 | 11,053 (14.8%) | 2,431 (15.6%) | 1,125 (20.6%) |
|  | 45-54 | 7,663 (10.3%) | 1,241 (7.9%) | 600 (11.0%) |
|  | $\geq$55 | 11,897 (16.0%) | 695 (4.4%) | 261 (4.8%) |
| Educational attainment^a^ | Less than primary level | 9,020 (12.1%) | 814 (5.2%) | 292 (5.4%) |
|  | Primary school level | 44,009 (59.0%) | 9,505 (60.8%) | 3,279 (60.2%) |
|  | Any secondary school level or higher | 19,530 (26.2%) | 4,090 (26.2%) | 1,213 (22.3%) |
| Occupation^b^ | Farmer | 36,691 (49.2%) | 6,072 (38.8%) | 2,330 (42.8%) |
|  | Student | 14,273 (19.1%) | 931 (6.0%) | 247 (4.5%) |
|  | Fishing, bar, or transportation | 5,425 (7.3%) | 3,426 (21.9%) | 1,102 (20.2%) |
|  | Other informal sector | 9,783 (13.1%) | 3,100 (19.8%) | 981 (18.0%) |
|  | Other formal sector | 3,366 (4.5%) | 814 (5.1%) | 203 (3.7%) |
|  | Unemployed or disabled | 4,270 (5.7%) | 835 (5.3%) | 218 (4.0%) |
|  | Other or unknown | 325 (0.4%) | 93 (0.6%) | 19 (0.3%) |
| Marital status^c^ | Not married | 22,123 (29.7%) | 4,265 (27.3%) | 1,053 (19.3%) |
|  | Married (monogamous) | 35,850 (48.1%) | 7,463 (47.7%) | 2,618 (48.1%) |
|  | Married (polygamous) | 8,001 (10.7%) | 2,374 (15.2%) | 960 (17.6%) |
|  | Divorced, separated, or widowed | 8,144 (10.9%) | 6,738 (43.1%) | 469 (8.6%) |
| Serodifferent partner | Yes | 1,815 (2.4%) | 1,815 (11.6%) | 1,026 (18.8%) |
|  | No or unknown | 72,726 (97.6%) | 13,817 (88.4%) | 4,421 (81.2%) |
| Circumcision^d^ | Medical | 7,498 (21.9%) | 2,054 (25.7%) | 742 (26.8%) |
|  | Traditional | 6,022 (17.6%) | 1,304 (16.3%) | 452 (16.3%) |
|  | Uncircumcised | 19,355 (56.7%) | 3,907 (48.8%) | 1,241 (44.8%) |
| Alcohol use^e^ | None | 62,923 (84.4%) | 11,840 (75.7%) | 3,896 (71.5%) |
|  | 1-7 days per month | 3,982 (5.3%) | 1,191 (7.6%) | 357 (6.6%) |
|  | >7 days per month | 5,696 (7.6%) | 1,388 (8.9%) | 536 (9.8%) |
| Mobility^f^ | Yes | 5,988 (8.0%) | 1,677 (10.7%) | 315 (5.8%) |
|  | No | 66,493 (89.2%) | 13,347 (85.4%) | 4,751 (87.2%) |
| Region | Western Kenya | 20,399 (27%) | 7,346 (47%) | 2,413 (44.3%) |
|  | Eastern Uganda | 26,923 (36%) | 3,876 (25%) | 1,471 (27.0%) |
|  | Western Uganda | 27,219 (37%) | 4,410 (28%) | 1,563 (28.7%) |

a. Missing data for 1,982 (2.7%) individuals.

b. Other formal sector occupations: teaching, government, military, health care, and factory work. Other informal sector occupations: shopkeeper, market vendor, hotel worker, homemaker, household worker, miner, and construction. Missing data for 408 (0.5%) individuals.

c. Missing data for 410 (0.6%) individuals.

d. Among men. Missing data for 1,265 (3.7%) individuals.

e. Missing data for 1,940 (2.6%) individuals.

f. Mobility defined as migration out of the community for at least 1 month or moved residence within the past 12 months.
