# Supplementary material for: HIV incidence after pre-exposure prophylaxis initiation among women and men at elevated HIV risk: A population-based study in rural Kenya and Uganda
Source: PLoS Med. 2021 Feb 9;18(2):e1003492. doi: 10.1371/journal.pmed.1003492 (PMC7872279; doi:10.1371/journal.pmed.1003492)
Supplement: S3 Table — PrEP, pre-exposure prophylaxis. (DOCX) [file pmed.1003492.s009.docx]

**S3 Table. Baseline characteristics of women and men who initiated PrEP in 16 communities in rural Kenya and Uganda**

|  |  | **PrEP initiators**  **(N = 5,447)** | **Women**  **(N = 2,674)** | **Men**  **(N = 2,773)** |
| --- | --- | --- | --- | --- |
| Age, years | 15-24 | 1,582 (29.0%) | 746 (27.9%) | 836 (30.1%) |
|  | 25-34 | 1,879 (34.5%) | 973 (36.4%) | 906 (32.7%) |
|  | 35-44 | 1,125 (20.6%) | 603 (22.6%) | 522 (18.8%) |
|  | 45-54 | 600 (11.0%) | 289 (10.8%) | 311 (11.2%) |
|  | $\geq$55 | 261 (4.8%) | 63 (2.4%) | 198 (7.1%) |
| Educational attainment^a^ | Less than primary level | 292 (5.4%) | 189 (7.1%) | 103 (3.7%) |
|  | Primary school level | 3,279 (60.2%) | 1,760 (6.6%) | 1,519 (5.5%) |
|  | Any secondary school level or higher | 1,213 (22.3%) | 394 (14.7%) | 819 (29.5%) |
| Occupation^b^ | Farmer | 2,330 (42.8%) | 1,352 (50.6%) | 978 (35.3%) |
|  | Student | 247 (4.5%) | 81 (3.0%) | 166 (6.0%) |
|  | Fishing, bar, or transportation | 1,102 (20.2%) | 289 (10.8%) | 813 (29.3%) |
|  | Other informal sector | 981 (18.0%) | 510 (19.1%) | 471 (17.0%) |
|  | Other formal sector | 203 (3.7%) | 85 (3.2%) | 118 (4.3%) |
|  | Unemployed or disabled | 218 (4.0%) | 145 (5.4%) | 73 (2.6%) |
|  | Other or unknown | 19 (0.3%) | 4 (0.1%) | 15 (0.5%) |
| Marital status^c^ | Not married | 1,053 (19.3%) | 248 (9.3%) | 805 (29.0%) |
|  | Married (monogamous) | 2,618 (48.1%) | 1,285 (48.1%) | 1,333 (48.1%) |
|  | Married (polygamous) | 960 (17.6%) | 622 (23.3%) | 338 (12.2%) |
|  | Divorced, separated, or widowed | 469 (8.6%) | 311 (11.6%) | 158 (5.7%) |
| Serodifferent partner | Yes | 1,026 (18.8%) | 620 (23.2%) | 406 (14.6%) |
|  | No or unknown | 4,421 (81.2%) | 2,054 (76.8%) | 2,367 (85.4%) |
| Circumcision^d^ | Medical | NA | NA | 742 (26.8%) |
|  | Traditional | NA | NA | 452 (16.3%) |
|  | Uncircumcised | NA | NA | 1,241 (44.8%) |
| Alcohol use^e^ | None | 3,896 (71.5%) | 2,205 (82.5%) | 1,691 (61.0%) |
|  | 1-7 days per month | 357 (6.6%) | 85 (3.2%) | 272 (9.8%) |
|  | >7 days per month | 536 (9.8%) | 57 (2.1%) | 479 (17.3%) |
| Mobility^f^ | Yes | 315 (5.8%) | 88 (3.3%) | 227 (8.2%) |
|  | No | 4,751 (87.2%) | 2,366 (88.5%) | 2,385 (86.0%) |
| Region | Western Kenya | 2,413 (44.3%) | 1,261 (47.2%) | 1,152 (41.5%) |
|  | Eastern Uganda | 1,471 (27.0%) | 735 (27.5%) | 736 (26.5%) |
|  | Western Uganda | 1,563 (28.7%) | 678 (25.4%) | 885 (31.9%) |

a. Missing data for 663 (12.2%) individuals, 331 (12.4%) women, and 332 (12.0%) men.

b. Other formal sector occupations: teaching, government, military, health care, and factory work. Other informal sector occupations: shopkeeper, market vendor, hotel worker, homemaker, household worker, miner, and construction. Missing data for 347 (6.4%) individuals, 208 (7.8%) women ,and 139 (5.0%) men.

c. Missing data for 347 (6.3%) individuals, 208 (7.8%) women, and 139 (5.0%) men.

d. Assessed among 2,773 men. Missing data for 338 (12.2%).

e. Missing data for 658 (12.1%) individuals, 327 (12.2%) women, and 331 (11.9%) men.

f. Mobility defined as migration out of the community for at least 1 month or moved residence within the past 12 months. Missing data for 381 (7.0%) individuals, 220 (8.2%) women, and 161 (5.8%) men.
