# Supplementary material for: HIV incidence after pre-exposure prophylaxis initiation among women and men at elevated HIV risk: A population-based study in rural Kenya and Uganda
Source: PLoS Med. 2021 Feb 9;18(2):e1003492. doi: 10.1371/journal.pmed.1003492 (PMC7872279; doi:10.1371/journal.pmed.1003492)
Supplement: S4 Table — PrEP, pre-exposure prophylaxis. (DOCX) [file pmed.1003492.s010.docx]

**S4 Table. Baseline characteristics of individuals who initiated PrEP and those with follow-up HIV testing after PrEP initiation**

|  |  | **All PrEP initiators**  **(n = 5,447)** | **Follow-up HIV testing**  **(n = 4,260)** |
| --- | --- | --- | --- |
| Sex | Female | 2,674 (49.1%) | 2,181 (51.2%) |
|  | Male | 2,773 (50.9%) | 2,079 (48.8%) |
| Age, years | 15-24 | 1,582 (29.0%) | 1,124 (26.4%) |
|  | 25-34 | 1,879 (34.5%) | 1,455 (34.2%) |
|  | 35-44 | 1,125 (20.7%) | 943 (22.1%) |
|  | 45-54 | 600 (11.0%) | 510 (12.0%) |
|  | $\geq$55 | 261 (4.8%) | 228 (5.4%) |
| Educational attainment^a^ | Less than primary level | 292 (5.4%) | 243 (5.7%) |
|  | Primary school level | 3,279 (60.2%) | 2653 (62.3%) |
|  | Any secondary school level or higher | 1,213 (22.3%) | 882 (20.7%) |
| Occupation^b^ | Farmer | 2,330 (42.8%) | 1,859 (43.6%) |
|  | Student | 247 (4.5%) | 189 (4.4%) |
|  | Fishing, bar, or transportation | 1,102 (20.2%) | 873 (20.5%) |
|  | Other informal sector | 981 (18.0%) | 741 (17.4%) |
|  | Other formal sector | 203 (3.7%) | 147 (3.5%) |
|  | Unemployed or disabled | 218 (4.0%) | 184 (4.3%) |
|  | Other or unknown | 19 (0.3%) | 13 (0.3%) |
| Marital status^c^ | Not married | 1,053 (19.3%) | 691 (16.2%) |
|  | Married (monogamous) | 2,618 (48.1%) | 2,140 (50.2%) |
|  | Married (polygamous) | 960 (17.6%) | 821 (19.3%) |
|  | Divorced, separated, or widowed | 469 (8.6%) | 354 (8.3%) |
| Serodifferent partner | Yes | 1,026 (18.8%) | 928 (21.8%) |
|  | No or unknown | 4,421 (81.2%) | 3,332 (78.2%) |
| Circumcision^d^ | Medical | 742 (26.8%) | 599 (28.9%) |
|  | Traditional | 452 (16.3%) | 372 (17.9%) |
|  | Uncircumcised | 1,241 (44.8%) | 887 (42.7%) |
| Alcohol use^e^ | None | 3,896 (71.5%) | 3,174 (74.5%) |
|  | 1-7 days per month | 357 (6.6%) | 243 (5.7%) |
|  | >7 days per month | 536 (9.8%) | 362 (8.5%) |
| Mobility^f^ | Yes | 315 (5.8%) | 200 (4.7%) |
|  | No | 4,751 (87.2%) | 3,785 (88.9%) |

a. Missing data for 663 (12.2%) individuals and 482 (11.3%) with follow-up HIV testing.

b. Other formal sector occupations: teaching, government, military, health care, and factory work. Other informal sector occupations: shopkeeper, market vendor, hotel worker, homemaker, household worker, miner, and construction. Missing data for 347 (6.4%) individuals and 254 (6.0%) with follow-up HIV testing.

c. Missing data for 347 (6.4%) individuals and 254 (6.0%) with follow-up HIV testing.

d. Among men. Missing data for 338 (12.2%) male individuals and 221 (10.6%) with follow-up HIV testing.

e. Missing data for 658 (12.1%) individuals and 481 (11.3%) with follow-up HIV testing.

f. Mobility defined as migration out of the community for at least 1 month or moved residence within the past 12 months. Missing data for 381 (7.0%) individuals and 275 (6.5%) with follow-up HIV testing.
