# Supplementary material for: HIV incidence after pre-exposure prophylaxis initiation among women and men at elevated HIV risk: A population-based study in rural Kenya and Uganda
Source: PLoS Med. 2021 Feb 9;18(2):e1003492. doi: 10.1371/journal.pmed.1003492 (PMC7872279; doi:10.1371/journal.pmed.1003492)
Supplement: S5 Table — PrEP, pre-exposure prophylaxis. (DOCX) [file pmed.1003492.s011.docx]

**S5 Table. Number of incident HIV infections, person-time at risk (in years), and HIV incidence rate (per 100 person-years) for PrEP initiators in all 16 study communities, PrEP initiators living in 8 communities where population-based HIV testing was conducted in the year prior to PrEP availability, and matched controls – overall and by sex.**

|  | **Incident HIV infections** | **Person-years** | **HIV incidence rate**  **per 100 person-years (95% CI)** |
| --- | --- | --- | --- |
| ***All 16 study communities*** |  |  |  |
| PrEP initiators | 25 | 7149.9 | 0.35 (0.22-0.49) |
| Women | 17 | 3735.2 | 0.46 (0.24-0.68) |
| Men | 8 | 3414.7 | 0.23 (0.09-0.41) |
|  |  |  |  |
| ***8 communities included in analysis of matched controls*** |  |  |  |
| PrEP initiators | 11 | 3393.3 | 0.32 (0.15-0.53) |
| Women | 7 | 1739.1 | 0.40 (0.12-0.73) |
| Men | 4 | 1654.3 | 0.24 (0.06-0.49) |
| Matched Controls | 17 | 1848.3 | 0.92 (0.49-1.41) |
| Women | 13 | 853.3 | 1.52 (0.70-2.36) |
| Men | 4 | 995.0 | 0.40 (0.10-0.90) |
